# Computer-Aided Drug Design for Type 2 Diabetes-Related Enzymes: SILCS-Based Fragment Mapping, Pharmacophore Modeling, and Virtual Screening

**DOI:** 10.1021/acs.jcim.6c01733

**Published:** 2026-09-30

**Authors:** Esmat Mohammadi, Justin A. Lemkul

**Affiliations:** † Department of Chemical Engineering, Virginia Tech, Blacksburg, Virginia 24061, United States; ‡ Department of Biochemistry, Virginia Tech, Blacksburg, Virginia 24061, United States; § Center for Computer-Aided Drug Design, Virginia Tech, Blacksburg, Virginia 24061, United States

## Abstract

Highly charged enzyme active sites present a challenge for conventional Site Identification by Ligand Competitive Saturation (SILCS) because strongly interacting charged probes can suppress sampling of neutral fragments, while additive force fields do not explicitly account for electronic polarization. To address these limitations, we developed a comprehensive SILCS-based computational framework and applied it to the N-terminal and C-terminal domains of maltase–glucoamylase and to α-amylase. Both the conventional mixed-probe SILCS workflow and a modified neutral–charged protocol were evaluated using the CHARMM additive and Drude polarizable force fields. FragMap convergence analysis demonstrated reliable sampling across all systems, with the neutral–charged protocol alleviating competitive exclusion effects observed in conventional simulations. In highly charged catalytic pockets, the conventional workflow was dominated by cationic probe sampling, suppressing neutral and hydrogen-bonding features. Separation of charged and neutral probes restored chemically diverse interaction patterns, improving representation of hydrogen-bond donor, acceptor, and hydrophobic hotspots. Incorporation of the Drude polarizable force field further enhanced resolution of electrostatic and hydrogen-bonding interactions. Energy-based SILCS-Pharmacophore models were generated from the neutral–charged FragMaps and used for virtual screening of the Maybridge and ChemBridge libraries. Hits were refined using SILCS-MC, ranked by ligand grid free energy (LGFE) and ligand efficiency (LE), and analyzed through scaffold- and fingerprint-based clustering. Integration of energetic and structural analyses enabled identification of favorable scaffolds and functional group patterns associated with strong binding efficiency. Together, this study develops, evaluates, and applies an improved SILCS workflow to three enzymes associated with type 2 diabetes (T2D), demonstrating its utility for characterizing highly charged active sites and prioritizing candidate inhibitors.

## Graphical abstract

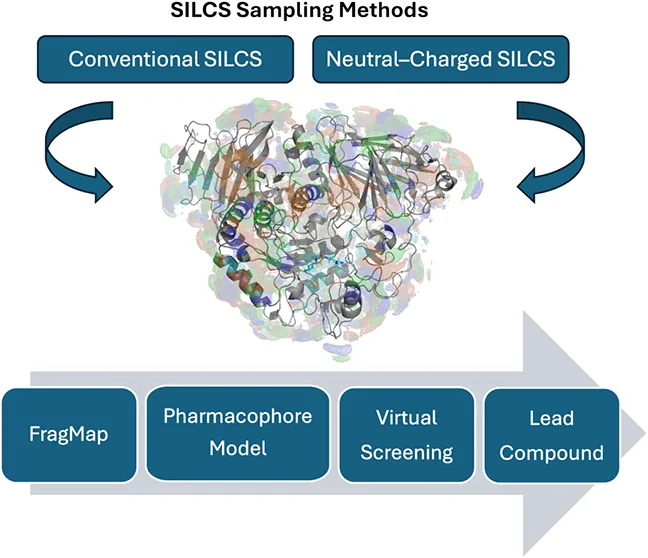

## Introduction

Type 2 diabetes (T2D) is a chronic metabolic disorder characterized by insulin resistance and impaired regulation of blood glucose levels. Persistent hyperglycemia is associated with severe long-term complications, including cardiovascular disease, neuropathy, nephropathy, and retinopathy.^1,2^ As the global prevalence of T2D continues to rise, there remains a strong need for effective therapeutic strategies that can provide sustained glycemic control with minimal adverse effects.^3,4^

Enzymes involved in carbohydrate digestion and glucose metabolism, such as the N- and C-terminal domains of maltase-glucoamylase (NtMGAM and CtMGAM, respectively), and pancreatic α-amylase, play a central role in postprandial glucose regulation and have therefore been widely explored as pharmacological targets for the treatment of T2D.^5,6^ Inhibition of these enzymes can delay carbohydrate digestion and reduce glucose absorption thereby lowering postprandial blood glucose levels. Although several enzyme inhibitors are currently used clinically, their application is often limited by insufficient selectivity, gastrointestinal side effects, or reduced long-term efficacy.^6,7^ These challenges motivate the continued search for novel inhibitors with improved binding profiles and optimized physicochemical properties, which may require new methodological advances, particularly within the domain of computer-aided drug design.

Structure-based drug design (SBDD) has become an essential tool in modern drug discovery. More broadly, structure-based computational methods are increasingly used to identify molecular recognition sites and support the design of therapeutic molecules.^8^ However, applying these approaches to enzymes involved in T2D presents several challenges. Many of these enzymes possess binding sites that are flexible, solvent-exposed, or extended, allowing them to accommodate a wide range of substrates.^9^ Such characteristics complicate the identification of high-affinity and selective small-molecule inhibitors.^10,11^

Traditional docking approaches often rely on a limited number of static protein conformations and simplified treatment of solvent effects.^12^ As a result, they may fail to capture induced-fit effects, transient binding pockets, or water-mediated interactions that are critical for ligand recognition.^13^ In addition, the energetic contribution of desolvation and competition between functional groups is frequently approximated rather than explicitly modeled.^14^ These limitations can lead to inaccurate ranking of ligands and reduced predictive power in virtual screening campaigns.

Site Identification by Ligand Competitive Saturation (SILCS) is a fragment-based computational approach designed to overcome many of the limitations associated with conventional docking and pharmacophore methods.^15^ SILCS employs all-atom molecular dynamics (MD) simulations in explicit solvent, in which a diverse set of small organic probe molecules is introduced at high concentration around the target protein.^16^ These probes competitively sample favorable interaction sites on the protein surface while accounting for protein flexibility, solvation, and entropic effects.^17^ From these simulations, three-dimensional fragment probability maps, known as FragMaps, are generated.^17^ FragMaps represent the spatial distribution and free-energy favorability of specific functional group interactions, such as hydrophobic, hydrogen-bond donor, hydrogen-bond acceptor, and charged interactions.^17^ Because FragMaps are derived from extensive MD sampling, they provide a thermodynamically grounded description of ligand–protein interactions that inherently incorporates conformational dynamics and solvent competition.^17^ Pharmacophore models can subsequently be generated from the FragMaps^18^ and ligands can be scored using a Ligand Grid Free Energy (LGFE) approach, which quantitatively estimates binding favorability based on overlap with the FragMaps.^19^

Although SILCS has a conventional workflow for protein systems using the CHARMM additive force field (FF),^19^ enzymes with highly charged active sites that are rich in charged residues may present a challenge. In the case of polyanionic RNA, positively charged fragments dominate the target biomolecule, effectively blocking neutral or less favorable fragments from adequately sampling the surface.^20^ The extent to which such behavior may occur in protein systems is unknown. Additionally, the use of a nonpolarizable FF in highly charged biomolecules may lack sensitivity given that this model does not account for environment-dependent changes in electronic distribution.^21^ Such polarization effects are especially important in highly charged or polar binding sites, where local electrostatic fluctuations strongly influence ligand binding.^22,23^

The Drude polarizable FF has previously been applied in a SILCS study to examine how electronic polarization influences protein–ligand interactions.^24^ Goel et al. showed that incorporating polarizability can improve the description of electrostatic interactions, leading to more favorable solute positioning and higher occupancy within known binding sites. However, prior work primarily focused on general protein systems and employed limited simulation replicates, without combining SILCS with enhanced sampling methods such as Grand Canonical Monte Carlo–MD (GCMC-MD). The absence of enhanced sampling can restrict the exploration of binding site regions, potentially underestimating the diversity of fragment interactions.

Here, we extend the SILCS framework to enzymes implicated in type 2 diabetes by integrating the Drude polarizable FF into a SILCS workflow that includes GCMC-MD sampling. This combined approach takes advantage of both explicit electronic polarization and the extensive fragment sampling achieved in the conventional SILCS simulations. By applying this polarizable SILCS workflow, we aim to capture the complex electrostatic environment of highly charged enzyme binding sites more accurately, improve the representation of fragment interactions, and enhance the predictive power of the resulting FragMaps and SILCS-Pharmacophore models. To assess the impact of explicit polarization on SILCS-derived FragMaps and Pharmacophore models, results obtained from the Drude-based workflow were also compared to those generated using the additive force field. In addition, in SILCS workflow we evaluated two sampling strategies, referred to as the “conventional” and “neutral-charged” approaches, in which neutral and charged probe molecules were simulated either together or separately to alleviate the exclusion of neutral fragments from highly polar and charged binding sites. This methodological adaptation provides a significant step toward more reliable computational screening of enzyme inhibitors in systems with highly charged or polar active sites. Accordingly, this study had two complementary objectives: to evaluate improved SILCS sampling strategies for highly charged protein systems and to apply the resulting models to the identification and prioritization of candidate inhibitors for three enzymes associated with type 2 diabetes.

## Methods

### Protein Preparation

The three-dimensional structures of the target enzymes were obtained from the RCSB Protein Data Bank.^25^ The selected systems included NtMGAM (PDB ID: 3L4Y), CtMGAM (PDB ID: 3TOP), and pancreatic α-amylase (PDB ID: 4GQR). The experimentally resolved structures are shown in Figure 1. Each structure was prepared and processed using the CHARMM package.^26^ Initial structure was subjected to energy minimization to relieve unfavorable steric contacts and optimize local geometry. Following minimization, all water molecules and ions were removed from the structures. The resulting minimized, stripped enzymes were then used as the starting configurations for the CHARMM-based SILCS simulations.

**Figure 1:**
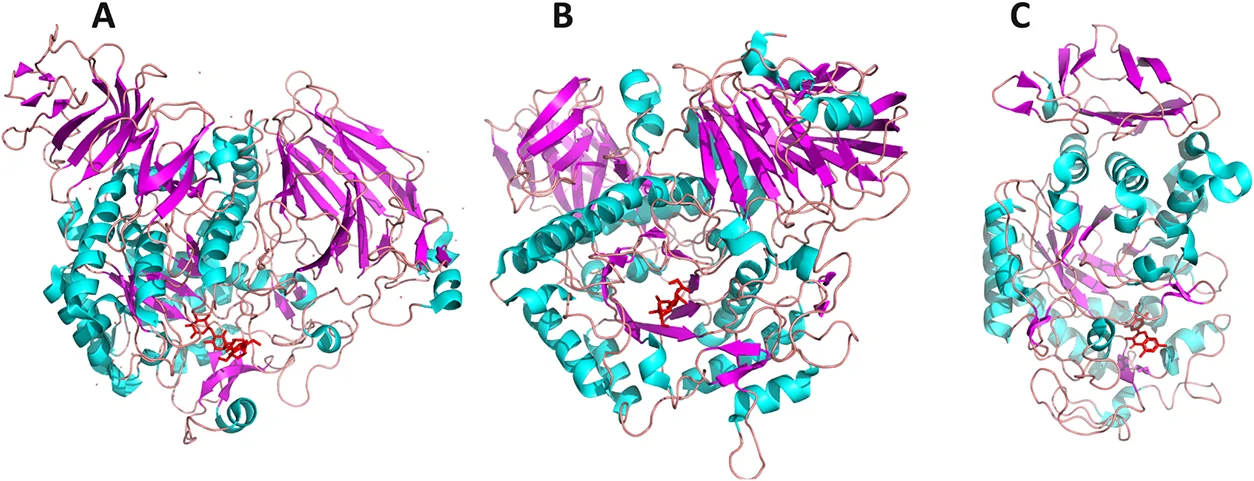
Crystal structures of (A) the N-terminal domain of maltase–glucoamylase, (B) the C-terminal domain of maltase–glucoamylase, and (C) α-amylase.

### CHARMM–SILCS Workflow: System Setup for Conventional Approach

For the conventional SILCS protein workflow, ten independent SILCS systems were constructed for each enzyme to ensure adequate statistical sampling.^16^ Each prepared enzyme structure was placed at the center of a rectangular simulation box with a minimum distance of 15 Å between the protein surface and the box boundaries in all directions. The systems were solvated with explicit water molecules, and a predefined set of small organic solutes was randomly distributed throughout the solvent to enable competitive fragment sampling. Water and solutes were added to concentrations of approximately 55 and 0.25 M, respectively.^27^ The solute mixture included both neutral and charged fragments including benzene, propane, dimethyl ether, methanol, formamide, imidazole, acetate, and methylammonium.^27^ For each enzyme, ten independent systems were generated using different random initial placements of solvent and solutes. These independently prepared systems served as the starting point for the CHARMM-based SILCS simulations. The conventional systems were subsequently subjected to GCMC refilling following the established SILCS protein workflow.^16,28,29^ Additional details of the conventional GCMC procedure are provided in the Supporting Information.

### CHARMM–SILCS Workflow: System Setup for Neutral–Charged Approach

In addition to the conventional SILCS workflow, a neutral–charged SILCS approach was employed to improve fragment sampling in highly charged enzyme binding sites. In this protocol, similar to the approach for SILCS-RNA protocol, the SILCS simulations were divided into two separate solute environments to mitigate competitive exclusion effects caused by strongly interacting charged fragments.^20^ One set of simulations contained only neutral solutes, including benzene, propane, dimethyl ether, methanol, formamide, and imidazole, while a second set contained only charged solutes, acetate and methylammonium.^20,29^

For each solute set, the target enzyme was placed at the center of a solvated simulation box with identical dimensions and solvent conditions as used in the conventional SILCS workflow, and ten independent systems were generated using randomized initial placements of solvent and solutes.^20^ By separating neutral and charged fragments into distinct simulations, this approach allows for more balanced sampling of diverse interaction types and ensures adequate exploration of both electrostatic and nonpolar interaction regions within the enzyme active sites. The resulting data from these simulations were subsequently combined during FragMap generation to provide a comprehensive representation of binding site interactions.

For the neutral system, refilling of water and probe molecules was carried out in three stages to improve sampling of buried and less accessible binding sites. In the first stage, the larger neutral fragments benzene, propane, and imidazole were inserted at an elevated concentration of approximately 0.35 M to improve their sampling of buried or sterically restricted regions. In the second stage, the remaining neutral and polar fragments, FORM, MEOH, and DMEE, were added to a standard concentration of approximately 0.25 M. In the final stage, water molecules were inserted to reach a concentration of approximately 55 M.^20^ Each refilling stage consisted of 200,000 GCMC steps, with insertion, deletion, translation, and rotation moves accepted or rejected according to the Metropolis criterion using the appropriate excess chemical potentials.^20^ For the charged system, a two-stage refilling procedure was applied. In the first stage, the charged fragments ACEY and MAMY were inserted into the GCMC-active region to reach their target concentrations. In the second stage, water molecules were added to restore the solvent concentration to approximately 55 M.^20^

### GCMC–MD Simulations

After completion of the GCMC refilling procedure, each system was energy-minimized using the steepest descent algorithm and subsequently equilibrated for 100 ps under weak harmonic positional restraints applied to all protein non-hydrogen atoms. SILCS sampling was then carried out using iterative GCMC–MD cycles. A total of 100 GCMC-MD cycles were performed for each system, with each cycle consisting of four sequential steps. First, GCMC sampling of all fragment probes and water molecules was conducted for 200,000 steps to promote extensive solute exchange and sampling within the binding region. This was followed by an additional steepest descent minimization, limited to either 5000 steps or convergence to a force tolerance of 10 kJ mol^–1^ nm^–1^. The systems were then equilibrated for 100 ps prior to a 1 ns MD production run, during which weak harmonic position restraints were applied to Cα atoms to maintain the tertiary structure of the enzymes, using a force constant of 50.208 kJ·mol^–1^·nm^–2^. This approach resulted in a cumulative production simulation time of 1 μs per enzyme for the conventional CHARMM SILCS workflow. All CHARMM additive GCMC-MD simulations were performed using SilcsBio package version 2023.1,^30^ coupled with GROMACS version 2022.5.^31^ The conventional simulations followed the standard SILCS protein workflow, whereas the neutral–charged sampling strategy, previously established for SILCS-RNA, was extended and evaluated here for protein systems. The Drude polarizable SILCS workflow described below was implemented separately in this study and is not part of the standard SilcsBio 2023.1 protein workflow.

### Drude SILCS Workflow

To take advantage of the GCMC sampling performed in the CHARMM SILCS simulations, evenly spaced snapshots were selected from these simulations and used to initialize the Drude SILCS calculations. Specifically, ten snapshots were extracted from each independent CHARMM system at the end of SILCS cycles 10, 20, 30, 40, 50, 60, 70, 80, 90, and 100. As a result, each of the ten nonpolarizable systems contributed ten starting structures, yielding a total of 100 initial configurations for the Drude SILCS simulations.

To make the coordinates of each snapshot compatible with CHARMM, the GROMACS PDB files were converted into CHARMM coordinate (CRD) format using a custom Python script that renamed atoms and added segment identifiers according to the Drude FF conventions. Lone pairs were added to hydrogen-bond acceptors and negatively charged Drude oscillators were added to all non-hydrogen atoms. Water molecules (represented by the CHARMM-modified TIP3P model^32–34^ ) were replaced with the polarizable SWM4-NDP^35^ model, and the Drude-2019 FF^36^ was applied to build the complete Drude topology. The systems were then minimized in two steps: an initial 1000-step steepest-descent minimization, followed by 2000 steps of adopted-basis Newton-Raphson minimization with restraints on the heavy atoms. This procedure allowed the induced dipoles to relax in the presence of the fixed charges.

Equilibration was conducted for 1 ns under an NPT ensemble using OpenMM 7.7.0.^37^ The system temperature was maintained at 298 K with a Langevin thermostat^38^ using a friction coefficient of 5 ps^–1^, while pressure was regulated at 1 atm through a Monte Carlo (MC) barostat. To ensure stable treatment of electronic polarization, Drude oscillators were coupled to a separate thermostat set to 1 K with a friction coefficient of 20 ps^–1^, and a hard-wall constraint^39^ was applied to restrict Drude–atom bond lengths to within 0.2 Å, preventing polarization catastrophe.^39^ Nonbonded interactions were treated using the particle mesh Ewald method^40,41^ for electrostatics, with a real-space cutoff of 12 Å and a Fourier grid spacing of 1 Å, and the van der Waals potential was smoothly switched between 10 and 12 Å. During equilibration, position restraints of 500.0 kJ/(mol·nm^2^) were applied to all non-hydrogen atoms of the enzymes. In contrast, solvent molecules and other solute atoms remained unrestrained, allowing them to adapt to the restrained protein framework. Following equilibration, each system was simulated in OpenMM 7.7.0 ^37^ for 20 ns per initial snapshot, resulting in 200 ns of sampling per system and a cumulative simulation time of 2 μs for each enzyme system. The same weak position restraints were applied to Ca atoms as in the CHARMM simulations.

### FragMap Generation and Analysis

FragMaps were generated from the production trajectories of the CHARMM and Drude SILCS simulations to describe the spatial preferences of probe solutes around the protein. Each simulation box was divided into a three-dimensional grid with a voxel size of 1 Å^3^, and the frequency of solute atom occupancy within each voxel was calculated over all simulation frames.

As listed in Table S1, solute atoms were grouped into chemically similar types, and occupancy values were normalized using the total number of frames, the number of atoms for each atom type (*n*_atoms_), and the bulk occupancy (OCC_bulk_), which is the corresponding bulk fragment concentration relative to water. These normalized occupancies were converted into free-energy FragMaps using a Boltzmann transformation as shown in eqs 1 and 2.

1 $$P_{\mathrm{xyz}}=\frac{{\mathrm{OC}C}_{\mathrm{xyz}}}{n_{atoms}\times {OCC}_{bulk}}$$

2 $${GFE}_{\mathrm{xyz}}=-\mathrm{RT}\ln\left(P_{\mathrm{xyz}}\right)$$

where OCC_*xyz*_ is the occupancy of the selected solute atom type in the voxel at (*x*,*y*,*z*), *n*_atoms_ is the number of atoms assigned to that atom type in the corresponding probe molecule, OCC_bulk_ is the bulk occupancy, *P*_*xyz*_ is the normalized occupancy, *R* is the gas constant, *T* is the absolute temperature, and GFE_*xyz*_ is the grid free energy at the voxel at (*x*,*y*,*z*). FragMap convergence was assessed using an overlap coefficient (OC) that compares maps derived from independent subsets of simulations. Specifically, FragMaps obtained from the first half of the independent runs (simulations 1-5) were compared to those from the second half (simulations 6-10), providing a quantitative measure of sampling consistency. OC values are calculated using eq 3

3 $$OC=\Sigma_{i=1}^{N}\min\left(\frac{Q_{i}^{1}}{\Sigma_{j=1}^{N}Q_{i}^{1}};\frac{Q_{i}^{2}}{\Sigma_{j=1}^{N}Q_{i}^{2}}\right)$$

where *N* denotes the total number of voxels in the FragMaps, *Q*_*i*_^1^ and *Q*_*i*_^2^ represent the occupancy of voxel *i* calculated from simulations in groups 1 and 2, respectively. OC values range from zero, indicating no similarity, to one, indicating that the maps are identical. OC values between 0.6 and 0.8 are generally considered to reflect sufficient similarity across independent simulations. In addition, exclusion maps were generated to identify regions that were not accessible to either water or fragment probes, representing volumes occupied by the protein.

### SILCS-Monte Carlo Pose Generation, Refinement, and Analysis

SILCS-MC was used for ligand pose refinement and docking using the CHARMM- and Drude-derived FragMaps.^28,42^ Ligand atoms were assigned to SILCS atom classes based on CGenFF,^43^ and LGFE was used to score ligand–FragMap interactions.^15^ Additional details of the SILCS-MC scoring procedure are provided in the Supporting Information.

SILCS-MC sampling was performed using two protocols: pose refinement and exhaustive (docking) sampling. The pose refinement protocol was applied when a reasonable initial binding pose was available, enabling targeted optimization of ligand configurations near the starting structure. In this protocol, each Monte Carlo/Simulated Annealing (MC/SA) cycle consisted of 100 MC steps followed by 1000 simulated annealing steps, with ligand movements limited to translations of up to 0.5 Å, rotations up to 15°, and intramolecular dihedral rotations up to 180°. In contrast, the exhaustive (docking) protocol was used when no prior information about the binding pose was available, requiring extensive sampling of ligand position and conformation. In this approach, the ligand was reoriented at the beginning of each cycle and subjected to longer MC/SA sampling (10,000 MC and 40,000 simulated annealing steps per cycle), with larger allowed movements including translations of up to 1 Å, rotations up to 180°, and dihedral rotations up to 180°. In both protocols, ligand–protein interactions were evaluated using LGFE scoring throughout the sampling process, and the lowest-energy clustered poses were retained for further analysis.^42^

### SILCS Pharmacophore Modeling

SILCS-based pharmacophore models were generated using the SILCS-Pharm protocol implemented through the SILCS graphical user interface (SILCS-GUI).^18,44^ For this procedure, the initial structures from the SILCS GCMC-MD simulations, along with the corresponding FragMaps, were imported into the GUI for pharmacophore generation, which was restricted to a spherical region of interest centered on the binding site to ensure comprehensive coverage of relevant interaction regions. A sphere with a radius of 12 Å was applied to include all features spanning the full binding site. Within this region, SILCS-Pharm identifies favorable interaction sites using predefined GFE thresholds. Map-specific GFE thresholds of −1.0 kcal/mol for the generic apolar map, −0.8 kcal/mol for the generic donor and generic acceptor maps, and −1.2 kcal/mol for the methylammonium nitrogen and acetate oxygen maps were used. These thresholds determine which favorable voxels were included in subsequent clustering and pharmacophore-feature generation, following the established SILCS-Pharm procedure.^16^ The charged-map cutoff is more restrictive than those used for the donor and acceptor maps, thereby limiting broad ionic regions while retaining weaker, spatially localized hydrogen-bonding regions. The same settings were applied consistently to all enzyme systems and force fields. In this approach, pharmacophore features consisted of cationic, anionic, hydrogen bond donor and acceptor, aromatic, and hydrophobic interactions.

The SILCS-Pharm workflow partitions the selected region into 1 Å cubic voxels corresponding to the same chemical interaction type, which are subsequently grouped using a hierarchical clustering algorithm. Clustering is performed based on distance criteria specific to the interaction class, with hydrogen bond donor and acceptor voxels clustered using a 1 Å cutoff, while aromatic and aliphatic voxels are clustered using a larger distance threshold of 2.8 Å. In the current implementation, pharmacophore features are derived from the generic apolar, generic donor, generic acceptor, methylammonium nitrogen, and acetate oxygen FragMaps.

Following clustering, each group of voxels is converted into a FragMap feature, which represents both the spatial location and chemical identity of a preferred interaction site. These FragMap features are subsequently transformed into pharmacophore features suitable for virtual screening. When features from different interaction classes overlap spatially, they may be represented as a joint pharmacophore feature. Consequently, more than one feature of the same individual or joint interaction type may be generated when distinct favorable regions occur at different locations within the binding site. Each pharmacophore feature was assigned a feature grid free energy (FGFE) value, computed as the sum of the GFE values of all voxels comprising a given cluster, therefore providing a quantitative measure of the feature’s contribution to ligand binding (eq 4). Final pharmacophore features were selected based on their FGFE values and their proximity to the binding site.

4 $${FGFE}_{j}=\Sigma_{i=1}^{n}{\;GFE}_{ith\;voxel\;in\;jth\;cluster}$$

where FGFE*_j_* is the feature grid free energy of cluster *j*, GFE*_i_* is the grid free energy of voxel *i* within that cluster, and *n* is the total number of voxels in the cluster. To balance selectivity with screening efficiency, pharmacophore models were curated to include a limited number of features. Final models consisted of up to four key pharmacophore features to capture essential interactions within the core binding region. This feature count was chosen to avoid overly restrictive models while minimizing the likelihood of excessive false-positive hits. The finalized pharmacophore models were exported in PH4 format, containing the spatial coordinates of each feature, associated radii, and feature identities for use in downstream virtual screening workflows.

### Virtual Screening

Virtual screening was performed using the Pharmit web-based platform to identify compounds compatible with the generated pharmacophore models.^45^ Screening was performed against the Maybridge and ChemBridge databases, containing 53,231 and 1,240,308 unique compounds, respectively, represented by 74,593 and 2,164,721 conformers. Additional details of the Pharmer search procedure are provided in the Supporting Information. To preserve chemical diversity during the screening stage, no upper limit was imposed on the total number of matches. All selected candidate compounds were exported for subsequent docking and refinement.

### Scaffold- and Fingerprint-Based Clustering Analysis

To investigate structural trends among the top-ranked screening hits and to identify favorable molecular scaffolds and functional group patterns, clustering analyses were performed on compounds identified from the Maybridge and ChemBridge virtual screening campaigns. Two complementary clustering strategies were employed: scaffold-based clustering using Bemis–Murcko frameworks^46^ and fingerprint-based clustering using molecular similarity metrics. Integration of ligand efficiency (LE) scores with clustering results enabled identification of scaffolds and structural motifs associated with favorable binding energetics. LE was calculated by dividing the LGFE by the number of ligand non-hydrogen atoms.

Bemis–Murcko scaffold analysis was used to group ligands according to their core structural frameworks. The Bemis–Murcko method extracts the central molecular skeleton by removing side chains and retaining ring systems and linker atoms. This representation captures the fundamental scaffold architecture of a compound and enables identification of common structural backbones among chemically diverse molecules. For each ligand, the Bemis–Murcko scaffold was generated using the RDKit cheminformatics toolkit.^47^ Compounds sharing identical scaffold structures were grouped into clusters, with each cluster representing a distinct core framework.

In parallel with scaffold-based grouping, molecular similarity clustering was performed using ligand fingerprints. For each ligand, Morgan fingerprints^48,49^ with a radius of two bonds (corresponding to atom environments extending up to two bonds from each atom) were generated as 2048-bit vectors to encode local structural features. Dimensionality reduction was applied using Uniform Manifold Approximation and Projection (UMAP)^50^ to project high-dimensional fingerprint data into a low-dimensional space suitable for clustering and visualization. Agglomerative hierarchical clustering was then performed on the reduced representation to group ligands based on structural similarity.^51^ To determine an optimal clustering threshold, multiple distance thresholds were evaluated, and clustering quality was assessed using the silhouette score.^52^ The threshold producing the highest silhouette score was selected for final cluster assignment.

To relate structural features to binding performance, cluster assignments obtained from both scaffold-based and fingerprint-based analyses were combined with ligand efficiency data derived from SILCS-MC refinement. Within each cluster, LE values were analyzed to identify groups of compounds exhibiting consistently favorable binding efficiency. Statistical distributions of LE values were evaluated to distinguish high-performing clusters from less favorable ones. Clusters with high average LE values and narrow distributions were interpreted as containing structurally promising scaffolds and functional group arrangements. These clusters were subsequently examined to identify common chemical motifs, including recurring hydrogen-bond donors and acceptors, charged functionalities, and hydrophobic elements.

The combined scaffold and fingerprint clustering framework enabled systematic identification of molecular frameworks associated with enhanced binding performance. Bemis–Murcko analysis highlighted dominant core structures, while fingerprint clustering provided finer resolution of substituent patterns and functional group variations. By correlating cluster membership with LE values, this workflow can facilitate prioritization of scaffolds and structural motifs for further optimization. The resulting analysis provides insight into supports rational design of improved inhibitors.

## Results and Discussion

### Convergence of the SILCS Simulations

Two SILCS sampling strategies were assessed in this study: a conventional protocol in which neutral and charged probes were simulated simultaneously, and a neutral–charged protocol in which neutral and charged fragments were sampled in separate environments. The primary metric for assessing convergence is the OC, which reflects the similarity of the maps generated across independent simulations. For the conventional SILCS workflow, both CHARMM and Drude simulations exhibited overall good convergence across all three enzyme systems (Table S2). In the CHARMM simulations, all OC values were ≥0.74 and most probe molecules in the Drude simulations had OC values on the order of 0.8. However, the charged acetate-carbon map had a lower OC (0.71–0.74) in the Drude simulations, suggesting increased sensitivity of ionic probes to electrostatic competition and local binding-site heterogeneity when neutral and charged fragments were sampled simultaneously. Taken together, the conventional workflow generated reproducible FragMaps for all three enzymes, NtMGAM, CtMGAM, and pancreatic α-amylase, with slightly reduced convergence for certain charged probes in the polarizable simulations.

To reduce competitive exclusion effects, a neutral–charged SILCS protocol was employed in which neutral and charged probes were simulated in separate environments. In the neutral-only simulations, CHARMM-based FragMaps exhibited convergence comparable to the conventional workflow, with OC values for most fragment types in the range of 0.74–0.77 (Table S3), and slightly higher OC values for hydrophobic probes. The corresponding Drude neutral simulations exhibited notably strong convergence, particularly for the α-amylase system. Several neutral and hydrophobic fragment types showed OC values exceeding 0.90 (Table S3), indicating consistent sampling over the surface of the enzymes. This improvement suggests that separating neutral fragments from charged probes enhances sampling efficiency and consistency in the polarizable simulations. In the charged-only simulations, CHARMM-based maps showed OC values between 0.71 and 0.83 for charged fragment types (Table S3), while in Drude charged simulations most charged probes produced OC values in the range of 0.76–0.83.

Across all enzymes and fragment types, both SILCS workflows produced FragMaps with reasonable convergence. The neutral–charged protocol preserved the reproducibility observed in the conventional workflow and, in several cases, improved convergence, particularly for neutral fragments in the Drude simulations. Charged FragMaps consistently exhibited slightly lower OC values than neutral or solvent maps, highlighting the intrinsic sensitivity of ionic probes to local electrostatic fluctuations. The OC values demonstrate reproducible FragMap sampling, while the comparisons of the FragMaps with the crystallographic binding poses of miglitol, acarbose, and myricetin discussed below provide a complementary assessment of their chemical relevance within the binding sites.

### Affinity Patterns for NtMGAM

To assess how fragment-sampling strategy and FF choice influence binding-site characterization, FragMaps generated for NtMGAM were compared between the conventional SILCS workflow (neutral and charged probes sampled together) and the neutral–charged workflow (neutral and charged probes simulated separately and combined during map construction). Using the conventional workflow, active-site FragMaps generated using the CHARMM FF were dominated by the methylammonium nitrogen map (Figure 2A), indicating that the positively charged probe preferentially occupied the pocket under mixed-probe conditions. As a result, hydrogen-bond donor, hydrogen-bond acceptor, and apolar features were largely excluded from the binding site. This pattern is consistent with competitive exclusion in a highly charged pocket; that is, strong electrostatic attraction of the cationic fragment out-competes weaker neutral interactions and limits the ability of other probes (apolar, donor, acceptor) to populate the site. For the Drude polarizable simulations, methylammonium nitrogen was also the dominant feature in the binding pocket (Figure 2C); however, unlike CHARMM, the conventional Drude maps exhibited contributions from additional interaction types. Apolar, hydrogen-bond donor, hydrogen-bond acceptor, and acetate carboxylate carbon features were detectable at lower occupancy around the binding-site region. This result suggests that explicit polarization partially moderates the flooding of the cationic probe and permits sampling by other fragments even under mixed conditions.

**Figure 2:**
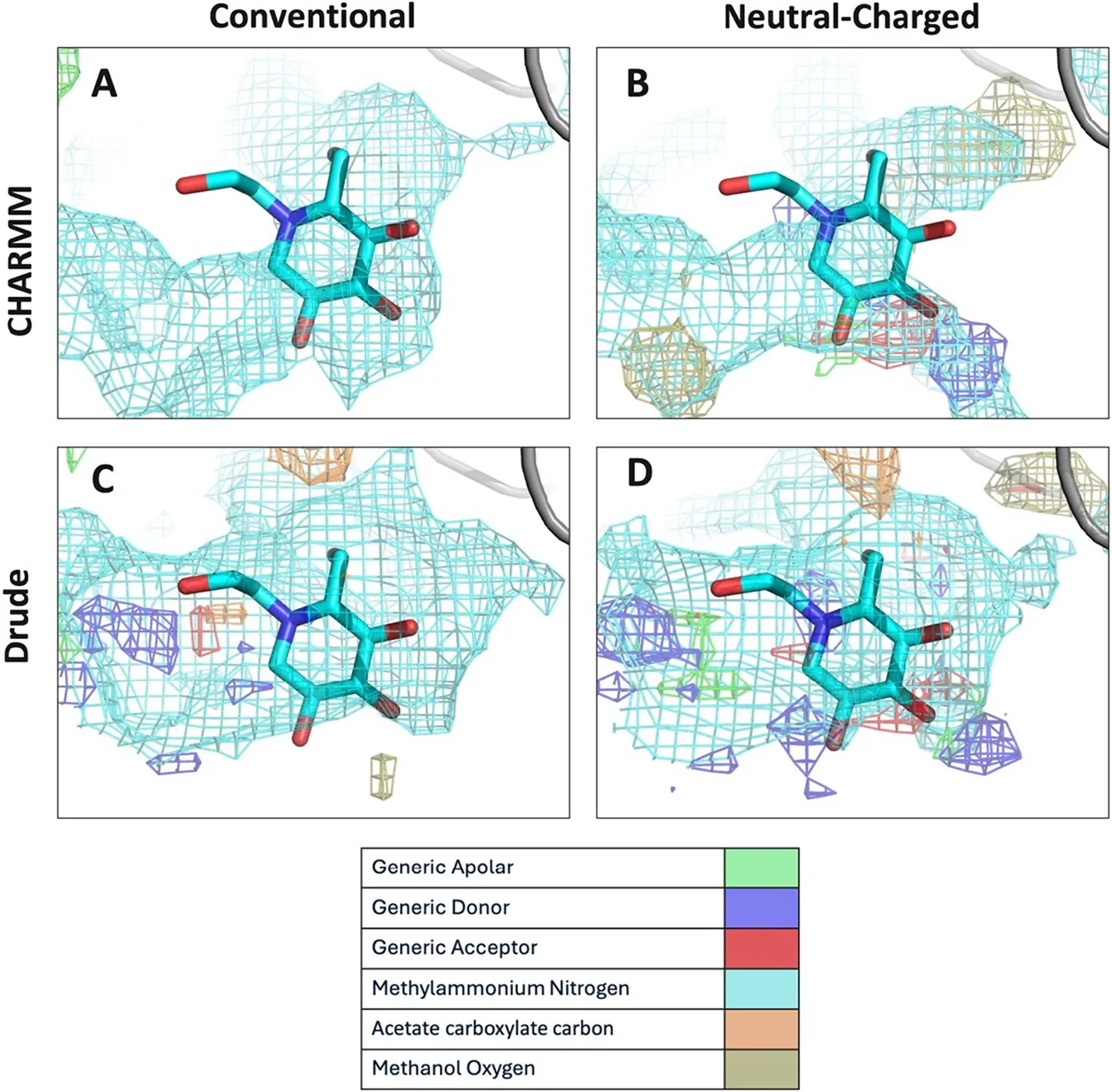
FragMaps identified for NtMGAM using the CHARMM FF with (A) conventional and (B) neutral–charged workflows, and using the Drude polarizable force field with (C) conventional and (D) neutral–charged workflows. The experimental binding pose of miglitol is shown in each panel for reference. The FragMaps are rendered at a GFE threshold of −1.2 kcal/mol.

In contrast, the neutral–charged CHARMM protocol produced a more diverse set of chemical features in the binding site (Figure 2B). In addition to methylammonium nitrogen, the maps show a clear presence of apolar, generic donor, generic acceptor, and methanol oxygen features. Importantly, these neutral/polar FragMaps overlapped with the known binding pose of miglitol, which is stabilized by hydrogen bonding to Asp, His, and Arg residues. In this workflow, separating charged and neutral fragments enabled neutral probes to sample the active site without being displaced by the strongly interacting cationic probe, allowing the FragMaps to better reflect the nature of carbohydrate-like inhibitor binding. Applying the neutral–charged protocol with the Drude FF led to a clear enrichment of non-cationic interaction maps in the binding site (Figure 2D). Although methylammonium sampling remained prominent, there was an increase in apolar, donor, and acceptor sampling, along with a greater contribution from the acetate carboxylate carbon map. This outcome indicates a more balanced exploration of the active site by both charged and neutral probes when they are sampled separately. The resulting FragMaps therefore suggest functional group diversity that may benefit inhibitor design, while still capturing the strong preference for cationic moieties in the active site.

### Affinity Patterns for CtMGAM

In the conventional CHARMM simulations of CtMGAM, the active site was principally occupied by the methylammonium nitrogen FragMap, indicating strong preferential sampling by the positively charged probe (Figure 3A). In addition, small regions of generic apolar density were observed in the active site. However, hydrogen-bond donor, hydrogen-bond acceptor, and methanol oxygen maps were effectively absent. This pattern suggests that strong electrostatic interactions dominate fragment sampling under mixed-probe conditions, suppressing weaker polar interactions, as was the case with NtMGAM. In the Drude conventional simulations, the active site was again dominated by methylammonium nitrogen FragMap (Figure 3C). However, compared with CHARMM, additional fragment types were present at low occupancy, including generic apolar, hydrogen-bond donor, acetate carboxylate carbon, and very weak methanol oxygen features. This result indicates that, like NtMGAM, explicit electronic polarization mitigates the strong attraction for cationic groups, permitting sampling by non-cationic probes even in the mixed environment.

**Figure 3:**
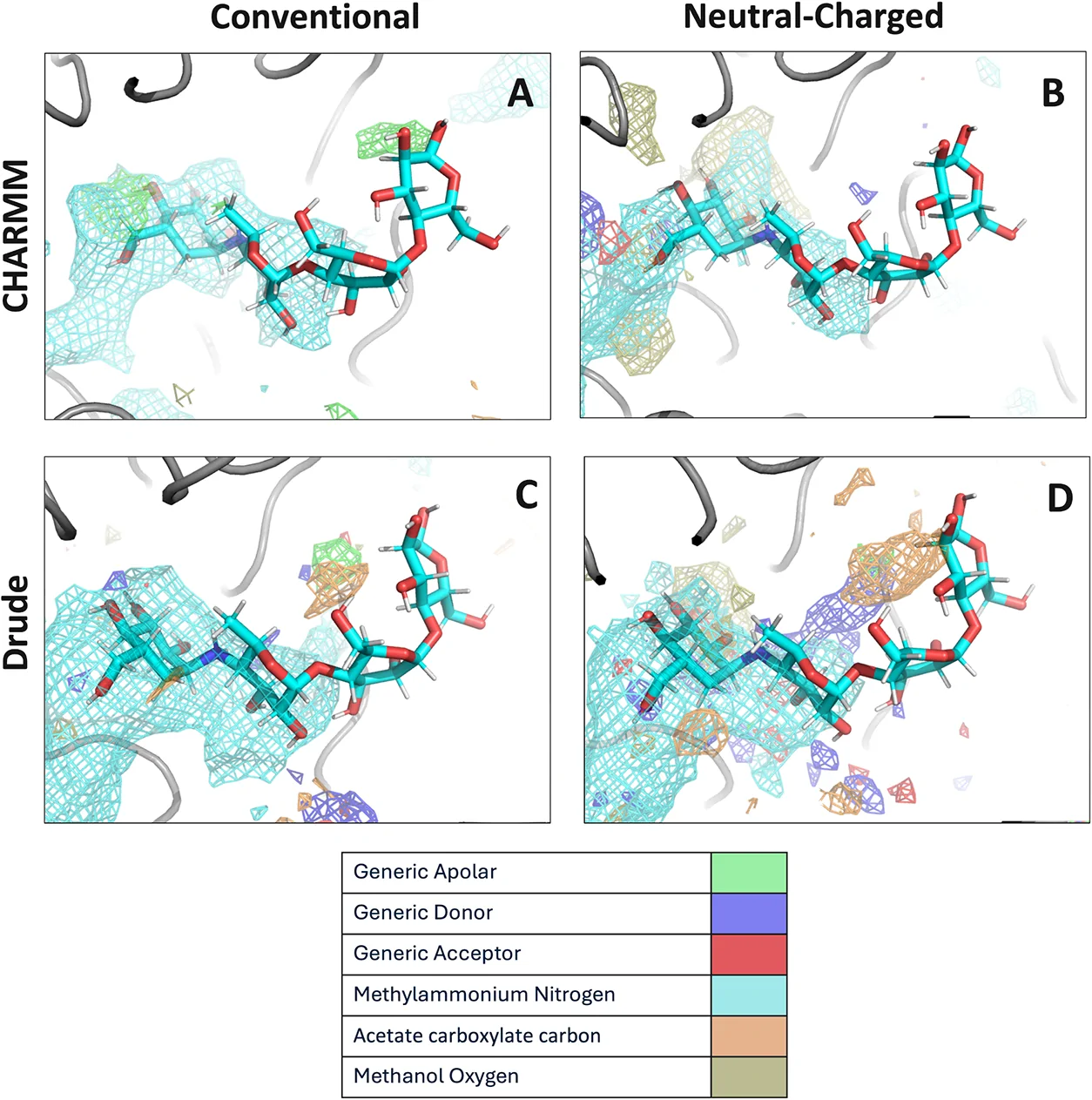
FragMaps identified for CtMGAM using the CHARMM FF with (A) conventional and (B) neutral–charged workflows, and using the Drude polarizable FF with (C) conventional and (D) neutral–charged workflows. The experimental binding pose of acarbose is shown in each panel for reference. The FragMaps are rendered at a GFE threshold of −1.2 kcal/mol.

The neutral–charged CHARMM simulations produced a richer set of FragMaps in the active site (Figure 3B). Alongside methylammonium nitrogen, clear hydrogen-bond donor and acceptor features were observed, along with methanol oxygen FragMaps. These polar interaction sites spatially overlap the crystal pose of the acarbose inhibitor. Importantly, previous experimental studies have identified hydrogen bonds with Asp and His residues, as well as hydrophobic contacts involving Trp, Phe, and Pro, are essential for acarbose binding.^53^ The presence of donor, acceptor, and apolar FragMaps in the neutral–charged simulations is therefore consistent with the known interaction network and indicates improved representation of functionally relevant binding features.

The conventional simulations with the Drude FF resulted in FragMaps that resembled those of the CHARMM FF, with a small region of acetate carboxylate carbon sampling (Figure 3C). Application of the neutral–charged protocol with the Drude FF resulted in a marked enhancement of chemically diverse interaction features. While methylammonium nitrogen remained prominent, there was a substantial increase in hydrogen-bond donor and acceptor maps, together with more favorable acetate carboxylate carbon and methanol oxygen features than with the CHARMM force field (Figure 3D).

### Affinity Patterns for α-Amylase

As with NtMGAM and CtMGAM, in the conventional CHARMM simulations of α-amylase, the active site was dominated by the methylammonium nitrogen FragMap, with some contributions from generic apolar features (Figure 4A). A very small methanol oxygen FragMap was also present, coinciding with one hydroxyl groups on the myricetin inhibitor. However, hydrogen-bond donor and acceptor maps were largely absent from the active site. As with NtMGAM and CtMGAM, the cationic probe outcompeted neutral hydrogen-bonding fragments and therefore may lack some essential features of potential inhibitors. In the Drude polarizable simulations using the conventional workflow, the active site was similarly dominated by methylammonium nitrogen (Figure 4C). However, compared to the CHARMM conventional results, low occupancies of hydrogen-bond donor and acceptor and generic apolar FragMaps were also observed. These additional maps align with the active-site features of α-amylase, wherein carbohydrate-like inhibitors rely heavily on hydrogen bonding with polar amino acids. As with NtMGAM and CtMGAM, these results suggest the inclusion of electronic polarization allows these groups to successfully compete for occupancy in the active site.

**Figure 4:**
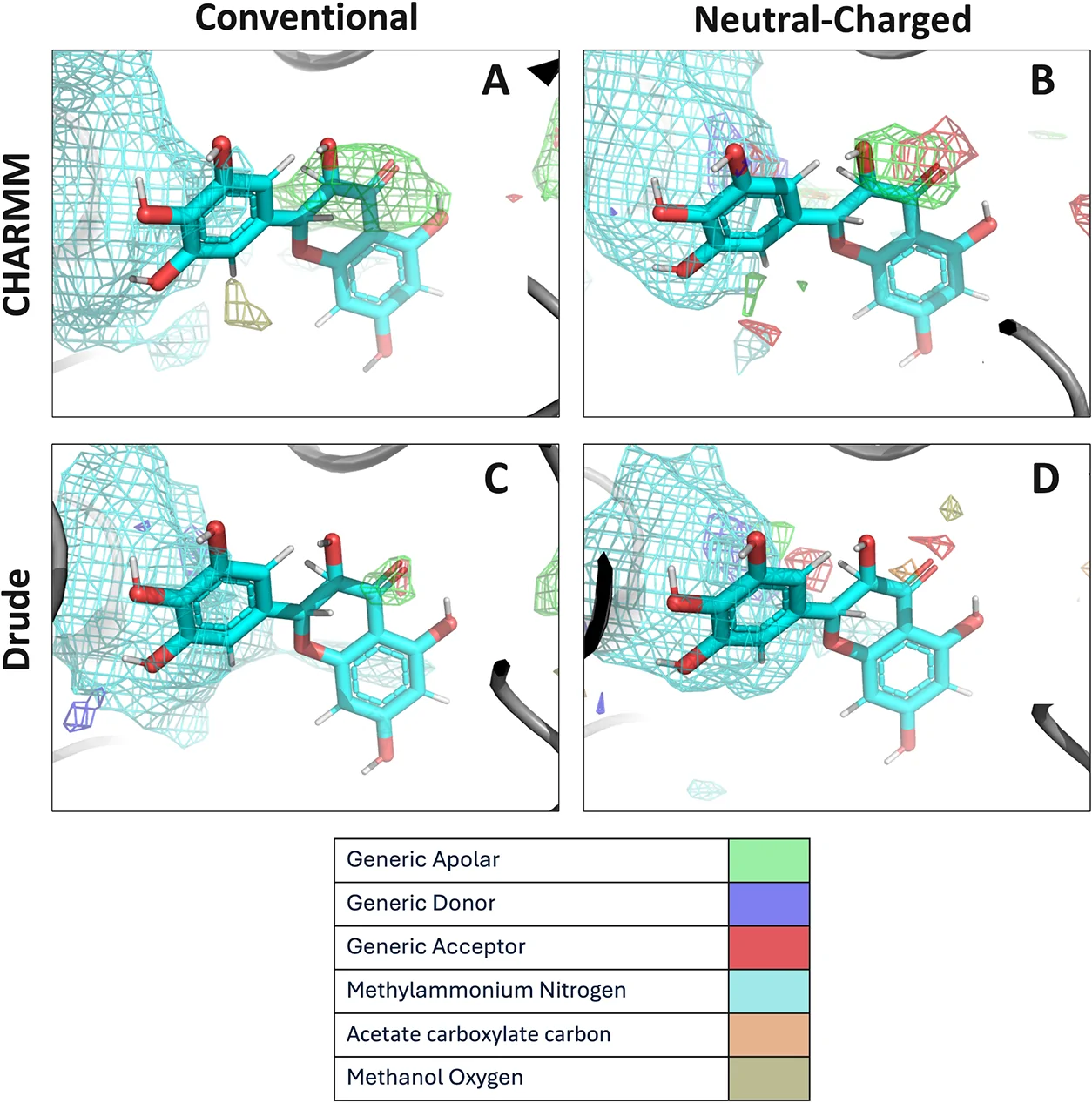
FragMaps identified for the α-amylase using the CHARMM FF with (A) conventional and (B) neutral–charged workflows and using the Drude polarizable FF with (C) conventional and (D) neutral–charged workflows. The experimental binding pose of myricetin is shown in each panel for reference. The FragMaps are rendered at a GFE threshold of −1.2 kcal/mol.

In contrast to the conventional approach, the neutral–charged CHARMM simulations yielded a more diverse set of FragMaps within the α-amylase active site (Figure 4B). In addition to methylammonium nitrogen and apolar features, small regions of apolar and hydrogen-bond donor and acceptor FragMaps were observed. The appearance of donor and acceptor FragMaps in regions overlapping the crystallographic myricetin ligand suggests improved representation of functionally relevant polar interactions when neutral and charged probes are sampled separately. Application of the neutral–charged protocol within the Drude framework resulted in a similar interaction landscape. Although methylammonium nitrogen remained the most prominent feature, there was an increase in hydrogen-bond donor and acceptor FragMaps compared to CHARMM (Figure 4D). The enhanced presence of donor and acceptor FragMaps indicates improved resolution of hydrogen-bonding interactions within the active site.

### Pose Refinement and Validation against Crystal Structures

To evaluate the accuracy of ligand pose prediction using the neutral-charged FragMaps generated with the CHARMM and Drude force fields, SILCS-MC local pose refinement was performed for known inhibitors using their crystallographic binding poses as starting structures. Specifically, miglitol was used for NtMGAM, acarbose for CtMGAM, and myricetin for α-amylase. Topologies for each inhibitor were generated with CGenFF^20^ and refinement was performed in the field of the FragMaps, attempting to maximize overlap between the ligand and the maps, driven by FragMap GFE values and augmented by an exclusion map representing inaccessible volume encompassing the enzyme. For each system, the lowest-energy poses obtained from SILCS-MC refinement were superimposed onto the corresponding crystal structures and visualized within the field of the CHARMM and Drude FragMaps (Figure 5). Structural agreement between refined and experimental poses was quantified by calculating the RMSD between heavy atoms of the crystallographic ligand and the SILCS-MC-refined conformation. Atom-wise GFE contributions for the SILCS-MC-refined poses are shown in Supporting Information, Figure S1. These values provide a direct visualization of the favorable and unfavorable contributions of individual ligand atoms within the corresponding FragMaps.

**Figure 5:**
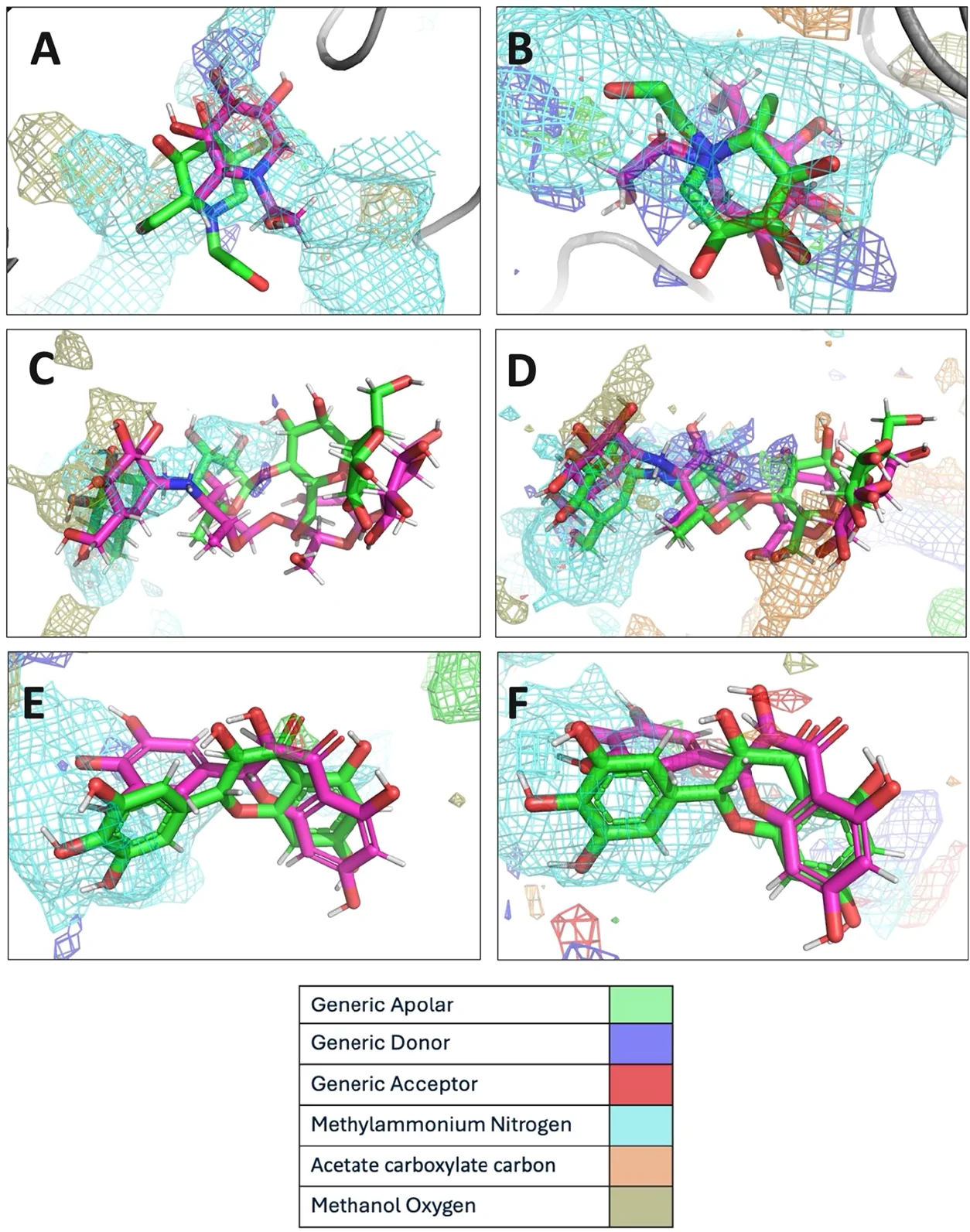
Comparison of (A) CHARMM- and (B) Drude-based pose refinement obtained from the neutral-charged FragMaps for miglitol in NtMGAM, (C) CHARMM- and (D) Drude-based pose refinement for acarbose in CtMGAM, and (E) CHARMM- and (F) Drude-based pose refinement for myricetin in α-amylase. In each panel, the crystallographic and SILCS-MC refined poses are represented by molecules with magenta and green carbon atoms, respectively.

For miglitol in NtMGAM, the CHARMM-based refinement produced a slightly higher RMSD (4.06 Å) than the Drude-based refinement (3.95 Å). Both the CHARMM and Drude FragMaps were dominated by methylammonium nitrogen density surrounding the ligand (Figure 5A,B). However, the Drude maps showed an increased hydrogen-bond donor density compared to those of CHARMM. Since miglitol contains multiple hydroxyl groups and a protonated amine capable of forming several hydrogen bonds, this increased presence of hydrogen-bond donors likely contributes to the improved pose agreement observed with Drude relative to CHARMM.

For acarbose in CtMGAM, pose refinement in the field of the Drude FragMaps resulted in substantially improved agreement with the crystallographic structure (RMSD = 1.30 Å) compared to CHARMM (RMSD = 3.38 Å). Both refined poses show overlap between the protonated nitrogen of acarbose and the methylammonium nitrogen features, as well as methanol oxygen features associated with hydroxyl groups (Figure 5C,D). The Drude FragMaps additionally display pronounced hydrogen-bond donor and acceptor regions that overlap with complementary polar groups on acarbose. The combined contributions of these chemically matched interactions may explain the more accurate reproduction of the experimental binding mode in the Drude framework.

For α-amylase, Drude FragMap-based pose refinement produced a lower RMSD (2.26 Å) than CHARMM (2.40 Å). In addition to the dominant methylammonium nitrogen features, the Drude FragMaps include hydrogen-bond donor and acceptor regions that overlap with polar functional groups on myricetin (Figure 5E,F). These additional interaction features likely contribute to more accurate pose prediction.

Taken together, these results indicate that SILCS-MC refinement using Drude FragMaps reproduces ligand poses more accurately than with the maps produced by the CHARMM FF, yielding better agreement with experimentally observed structures. This improvement, although modest in some cases, is likely due to the abundance of charged residues in the active site, whose electrostatic interactions are better captured by the Drude model. The inclusion of directional electrostatics in Drude FF enables a more realistic representation of these interactions.

### Pharmacophore Models for NtMGAM

Given the benefits of the neutral–charged workflow described above, pharmacophores were constructed for NtMGAM using FragMaps from the neutral–charged SILCS workflow using both CHARMM and Drude FFs. Pharmacophore features were identified based on spatial clustering of favorable grid free energy (GFE) regions and ranked according to their feature grid free energy (FGFE) values.^44^ Final pharmacophore models were selected based on a combination of FGFE ranking and spatial localization within the active site of each enzyme.

Using CHARMM-derived FragMaps, a total of 13 pharmacophore features were identified for NtMGAM, with FGFE values ranging from −96.17 to −1.15 kcal/mol (Table 1). The features with the most favorable FGFE values were those that included hydrogen-bond donors and cationic groups. The large methylammonium nitrogen FragMap contributed to the identification of several cationic donor features at different locations in the active site. These features were ranked using FGFE, which reflects both the energetic favorability and the size of each clustered region. Final selection also considered the location of each feature within the ligand-binding pocket. Spatial analysis revealed that some high-ranking features, including Feature 3, were located outside the primary ligand-binding pocket and therefore unlikely to participate directly in inhibitor recognition. To construct a chemically meaningful and spatially relevant model, final feature selection was based on both energetic and geometric criteria. As shown in Figure 6A and Table 2, the three selected features are positioned in the active site and are consistent with experimentally reported interactions involving hydrogen bonding to Asp, His, and Arg residues. The resulting pharmacophore model captures the dominant polar and electrostatic interactions required for inhibitor binding while avoiding over-restriction of the screening space.

**Figure 6:**
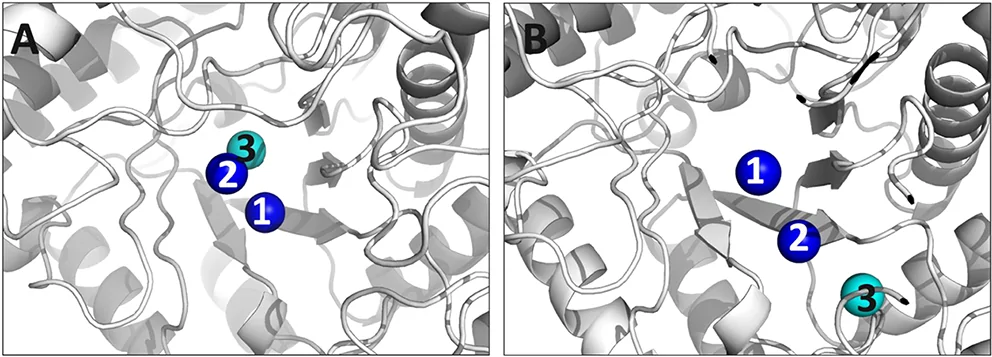
Selected pharmacophore features for NtMGAM generated using (A) CHARMM and (B) Drude FFs. Hydrogen-bond donor and cationic features are blue and cyan spheres, respectively.

**Table 1:** Complete List of Identified Pharmacophore Features for NtMGAM, CtMGAM, and α-Amylase, Generated from the Neutral-Charged FragMaps Obtained Using the CHARMM and Drude FFs^a^

	NtMGAM				CtMGAM				α-Amylase			
	CHARMM		Drude		CHARMM		Drude		CHARMM		Drude	
#	Feature	FGFE	Feature	FGFE	Feature	FGFE	Feature	FGFE	Feature	FGFE	Feature	FGFE
1	Don	–96.17	Don|Acc	–346.76	Don|Acc&!Ani	–90.01	Don|Acc&!Ani	–306.19	Don|Acc&!Ani	–156.35	Don|Acc&!Ani	–133.09
2	Don|Acc&!Ani	–94.95	Don	–71.61	Cat&Don	–41.67	Don&!Cat|Acc&!Ani	–19.74	Don|Acc&!Ani	–80.58	Don	–25.53
3	Cat&Don	–54.27	Don|Acc&!Ani	–71.21	Cat&Don	–12.36	Don&!Cat	–16.23	Aro|Hyd	–31.42	Don&!Cat	–16.08
4	Cat&Don	–52.91	Don&!Cat|Acc	–66.89	Don&!Cat	–8.23	Don&!Cat	–13.22	Cat&Don	–6.01	Aro|Hyd	–12.57
5	Cat&Don	–28.88	Aro|Hyd	–26.31	Cat&Don	–7.8	Cat&Don	–6.32	Cat&Don	–5.43	Don&!Cat|Acc&!Ani	–12.48
6	Cat&Don	–22.13	Cat&Don	–18.53	Don&!Cat	–3.4	Aro|Hyd	–4.99	Hyd	–4.05	Don&!Cat	–7.49
7	Cat&Don	–14.08	Cat&Don	–11.75	Don&!Cat	–3.16	Don&!Cat	–3.3	Aro	–3.78	Don&!Cat|Acc&!Ani	–7.02
8	Cat&Don	–11.37	Aro	–11.42	Acc&!Ani	–1.25	Acc&!Ani	–3.28	Acc&!Ani	–3.53	Acc&!Ani	–6.86
9	Cat&Don	–8.53	Don&!Cat	–10.9	Acc&!Ani	–1.24	Don&!Cat	–2.65	Acc&!Ani	–3.44	Acc&!Ani	–6.08
10	Aro	–5.1	Cat&Don	–9.14	Acc&!Ani	–1.12	Don&!Cat	–2.53	Acc&!Ani	–3.33	Acc&!Ani	–5.40
11	Aro	–4.98	Aro	–5.08	Acc&!Ani	–1	Acc&!Ani	–2.48	Aro	–2.45	Acc&!Ani	–5.02
12	Cat&Don	–2.27	Don	–4.88	Acc&!Ani	–1	Acc&!Ani	–2.12	Cat&Don	–1.84	Don&!Cat	–4.09
13	Acc&!Ani	–1.15	Acc&!Ani	–3.82			Don&!Cat	–2.1	Don&!Cat	–1.61	Hyd	–3.75
14			Ani&Acc	–3.82			Acc&!Ani	–2.06	Hyd	–1.21	Acc&!Ani	–2.30
15			Ani&Acc	–3.66			Cat&Don	–2.05	Acc&!Ani	–1.13	Acc&!Ani	–2.19
16			Acc&!Ani	–3.59			Cat&Don	–2.01	Acc&!Ani	–1.05	Don&!Cat	–2.19
17			Cat&Don	–2.28			Cat&Don	–1.97	Acc&!Ani	–1	Acc&!Ani	–2.09
18			Don&!Cat	–2.12			Acc&!Ani	–1.3			Acc&!Ani	–1.41
19			Cat&Don	–2.01			Don&!Cat	–1.25			Acc&!Ani	–1.33
20			Acc&!Ani	–1.26			Acc&!Ani	–1.06			Acc&!Ani	–1.28
21			Don&!Cat	–1.23			Acc&!Ani	–1.02			Don&!Cat	–1.22
22			Acc&!Ani	–1.22			Acc&!Ani	–1			Acc&!Ani	–1.16
23											Acc&!Ani	–1.13
24											Don&!Cat	–1.11
25											Acc&!Ani	–1.06
26											Don&!Cat	–1.01

a Pharmacophore feature types are defined as follows: hydrogen bond donor (Don), hydrogen bond acceptor (Acc), cationic (Cat), anionic (Ani), hydrophobic (Hyd), and aromatic (Aro). All FGFE values are given in kcal/mol.

**Table 2:** Pharmacophore Features Identified for NtMGAM Using CHARMM and Drude FFs, along with Their Corresponding FGFE Values (kcal/mol)

	Type	FGFE	Type	FGFE
Number	CHARMM		Drude	
1	HB Donor	–96.17	HB Donor	–71.61
2	HB Donor	–94.95	HB Donor	–71.21
3	Cation	–11.37	Cation	–9.15

In comparison to the CHARMM results, the Drude simulations yielded a larger number of pharmacophore features. A total of 22 interaction features were identified (Table 1), reflecting the increased chemical diversity and improved resolution of interaction sites. The most energetically favorable feature (FGFE = −346.76 kcal/mol), resulting from hydrogen-bond donors or acceptors, indicates a polar interaction site that was not observed with comparable strength in the CHARMM simulations. After evaluating both energetic ranking and spatial positioning, three features were selected for constructing the final Drude-based pharmacophore model (Figure 6B and Table 2). All selected features are located within the active site and their spatial arrangement is compatible with hydrogen-bonding and other electrostatic interactions essential for ligand stabilization. Supporting Information, Table S4 illustrates the energetic and spatial properties of all candidate pharmacophore features generated for NtMGAM using the CHARMM additive and Drude polarizable force fields. The center-to-center distances between the selected features were 4.60, 4.77, and 5.20 Å for the CHARMM model and 2.79, 4.15, and 5.69 Å for the Drude model. All selected features had a tolerance radius of 1.50 Å.

### Pharmacophore Models for CtMGAM

In the case of CtMGAM, the CHARMM FragMaps led to the identification of 12 distinct pharmacophore features, spanning FGFE values from −90.02 to −1.00 kcal/mol (Table 1). Most of these features correspond to hydrogen-bonding and cation-associated interaction sites, reflecting the highly polar nature of the CtMGAM active site. Notably, features associated with generic hydrophobic interactions were absent, suggesting that nonpolar contacts play a limited role in ligand stabilization when described by the CHARMM FF. Therefore, three features including hydrogen bond donor and cation features, were chosen to construct the final CHARMM-based pharmacophore model (Figure 7A and Table 3).

**Figure 7:**
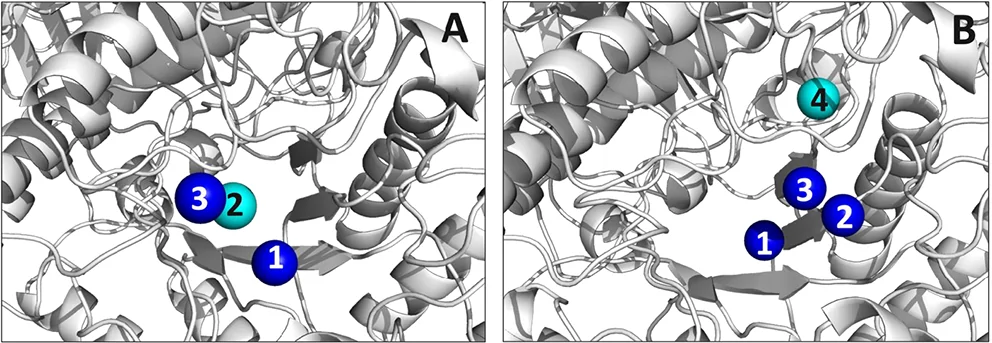
Selected pharmacophore features for CtMGAM generated using (A) CHARMM and (B) Drude FFs. Hydrogen-bond donor and cationic features are blue and cyan spheres, respectively.

**Table 3:** Pharmacophore Features Identified for CtMGAM Using CHARMM and Drude FFs, along with Their Corresponding FGFE Values (kcal/mol)

	Type	FGFE	Type	FGFE
Number	CHARMM		Drude	
1	HB Donor	–90.01	HB Donor	–306.19
2	Cation	–41.67	HB Donor	–19.74
3	HB Donor	–8.23	HB Donor	–13.22
4	-	-	Cation	–6.32

Application of the Drude polarizable FF resulted in identification of a more extensive and energetically diverse set of pharmacophore features. In total, 22 interaction sites were detected with FGFE values extending from −306.19 to −1.00 kcal/mol (Table 1). The most favorable interaction center (FGFE = −306.19 kcal/mol), reflects strong stabilization driven by polarization-enhanced hydrogen bonding and electrostatic interactions. Together, the feature sites define a network of favorable polar and ionic interaction regions within the active site. After assessing both energetic ranking and spatial correspondence, four hydrogen-bonding and cationic features were selected for inclusion in the final Drude-based pharmacophore model (Figure 7B and Table 3). The CHARMM- and Drude-derived pharmacophore models for CtMGAM highlight clear differences in how the C-terminal active site might be targeted. Although both CHARMM- and Drude-derived pharmacophores include both hydrogen-bond donors and cationic features, their spatial arrangement differs, particularly in the location of the cationic features (Figure 7). This difference likely arises from subtle differences in the FragMaps used to generate these pharmacophores (Figure 3), demonstrating the impact of explicit electronic polarization on the spatial arrangement of small-molecule chemical properties.

### Pharmacophore Model Generation for α-Amylase

Pharmacophore models for α-amylase were constructed from FragMaps obtained using the neutral–charged SILCS workflow with both CHARMM and Drude FFs and features selected in the same manner as NtMGAM and CtMGAM. Analysis of the CHARMM-based FragMaps resulted in the identification of 17 distinct pharmacophore features with FGFE values ranging from −156.36 to −1.00 kcal/mol (Table 1). These features represent a broad range of interaction types, including joint donor–acceptor sites, aromatic–hydrophobic regions, cation-associated donors, and multiple acceptor sites. Although several additional favorable features were generated from the FragMaps, they were located outside the active site and were therefore not considered further. Four features were selected to construct the final CHARMM pharmacophore model (Figure 8A and Table 4). Together, these pharmacophore features represent a combination of dominant hydrogen bonding contacts, including a cationic feature. Although the FragMaps indicated hydrophobic/aromatic occupancy of the active site, the most prominent features were still electrostatic in nature.

**Figure 8:**
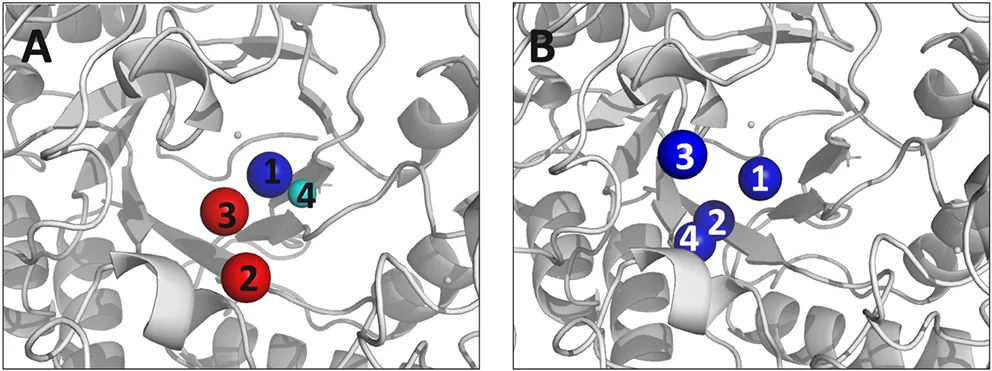
Selected pharmacophore features for the α-amylase generated using (A) CHARMM and (B) Drude FFs. Hydrogen-bond donor, hydrogen-bond acceptor, and cationic features are blue, red, and cyan spheres, respectively.

**Table 4:** Pharmacophore Features Identified for α-Amylase Using CHARMM and Drude FFs, along with Their Corresponding FGFE Values (kcal/mol)

	Type	FGFE	Type	FGFE
Number	CHARMM		Drude	
1	HB Donor	–156.35	HB Donor	–133.08
2	HB Acceptor	–3.53	HB Donor	–22.53
3	HB Acceptor	–3.33	HB Donor	–16.07
4	Cation	–1.84	HB Donor	–12.48

The use of the Drude FF led to identification of a larger and more heterogeneous set of interaction features than with the CHARMM FF. A total of 26 pharmacophore features were detected (Table 1), reflecting increased sensitivity to subtle electrostatic and hydrogen-bonding effects. The strongest interaction region (FGFE = −133.09 kcal/mol) corresponds to donor and acceptor features within the catalytic pocket. Several additional features associated with hydrogen-bond donors and acceptors were also favorable, indicating the presence of multiple polar interaction centers. Following evaluation of both FGFE ranking and spatial arrangement, four features were selected for construction of the final Drude pharmacophore model (Figure 8B and Table 4). These sites collectively describe a network of hydrogen-bonding interactions centered around the catalytic acidic residues. The Drude-derived pharmacophore relies entirely on hydrogen-bond donors, unlike the CHARMM-derived pharmacophore, which includes hydrogen-bond acceptors and cationic features. Thus, the two FFs lead to distinct pharmacophores that may predict very different small molecules as inhibitors. Although the methylammonium nitrogen FragMap was visually prominent, the cationic feature was not retained solely based on FragMap volume or probe frequency. Candidate features were evaluated based on their FGFE values and spatial locations within the catalytic site. The CHARMM cationic feature was retained despite its relatively weak FGFE of −1.84 kcal/mol because of its spatial relevance to the catalytic site. Thus, the CHARMM pharmacophore directly favored compounds with an appropriately positioned cationic center, whereas the absence of a cationic feature in the Drude pharmacophore resulted in a stronger emphasis on hydrogen-bond donor-rich compounds.

### Virtual Screening

Each pharmacophore model was employed independently to screen Maybridge and ChemBridge databases using the Pharmit Web server. Candidate compounds were retained if they satisfied the spatial and chemical constraints defined by the pharmacophore features and receptor-based shape filters. We performed additional SILCS-MC docking and ranked the compounds based on their LE scores. No overlap was identified between the Maybridge and ChemBridge hit sets.

### Virtual Screening and Lead Selection for NtMGAM

For NtMGAM, screening using CHARMM-derived pharmacophores yielded 311 hits from the Maybridge database and 100 hits from the ChemBridge database. In contrast, screening using Drude-derived pharmacophores resulted in 27 hits from Maybridge and 100 hits from ChemBridge. All compounds identified through pharmacophore screening were subjected to SILCS-MC docking to evaluate their compatibility using the generated FragMaps.

LGFE values were calculated for the lowest-energy poses. LE scores were subsequently computed to normalize predicted affinity to molecular size, and these LE values were used to rank compounds and enabled a systematic comparison across chemically diverse candidates. For each database and FF, the ten highest-ranking compounds based on LE scores were selected (Table S5). The most favorable LE score across all screened compounds from the Maybridge and ChemBridge databases was that of MAYBRIDGE-SEW03510-3, the structure of which is shown together with its lowest-energy binding pose obtained from SILCS-MC refinement in Figure 9. The compound contains a cationic heterocyclic ring that overlaps with the methylammonium nitrogen FragMap, indicating favorable electrostatic complementarity with the active site. The identification of the MAYBRIDGE-SEW03510-3 compound arose from the CHARMM FragMaps. Given the similarity of the two pharmacophores for NtMGAM with the two FFs, for this enzyme there may only be minor differences in performance between the CHARMM and Drude FFs and the nonpolarizable FF is sufficiently accurate to capture essential features for ligand design.

**Figure 9:**
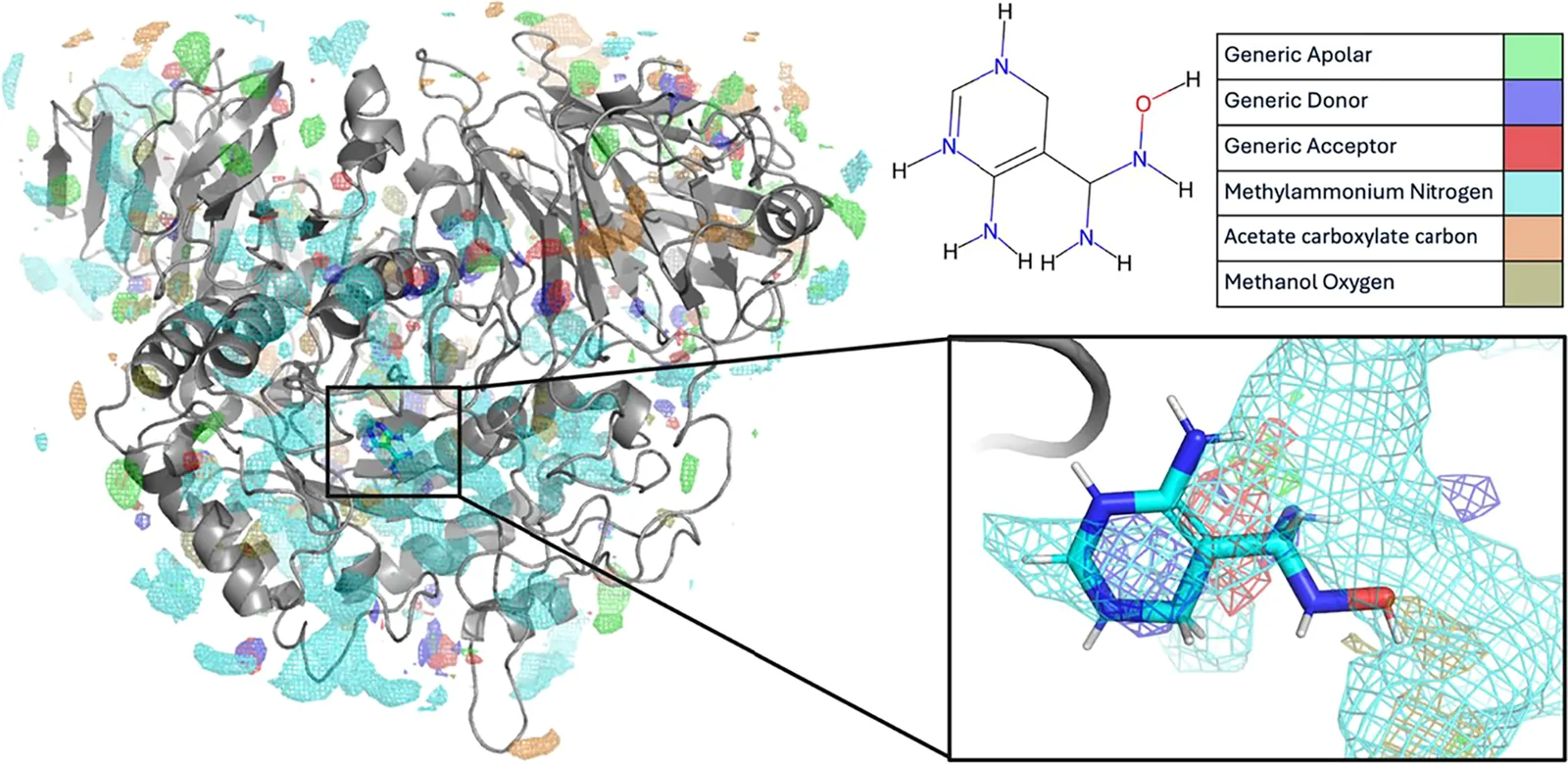
Chemical structure and lowest-energy binding pose of MAYBRIDGE-SEW03510-3 in NtMGAM. The SILCS-MC-refined pose is shown within the binding site and overlaid with CHARMM-derived FragMaps.

To further evaluate the predictive performance of the SILCS-based screening workflow, LGFE and LE scores were also calculated for known NtMGAM inhibitors (miglitol and acarbose) using SILCS-MC pose refinement. The resulting LGFE and LE values, together with those of the newly identified screening hits, are shown in Table S5 and were used as benchmarks for assessing relative binding strength. Analysis of the CHARMM-based results (Table S5) shows that miglitol exhibits a more favorable LGFE than acarbose, consistent with experimental studies reporting greater inhibitory potency of miglitol toward this domain. This agreement supports the reliability of the CHARMM-derived FragMaps and scoring framework for this system. In contrast, Drude-based calculations predict a more favorable LGFE for acarbose than for miglitol, although the LE value remains more favorable for miglitol. Despite this variation, MAYBRIDGE-SEW03510-3 exhibits substantially more favorable LGFE and LE values than either of the known inhibitors.

### Virtual Screening and Lead Selection for CtMGAM

For CtMGAM, application of the CHARMM pharmacophore resulted in identification of 30 candidate molecules from the Maybridge database and 100 candidates from the ChemBridge database. In contrast, screening with the Drude pharmacophore produced only one hit from Maybridge and 17 hits from ChemBridge. As shown above (Figure 7), the CHARMM and Drude pharmacophores were markedly different, particularly with respect to the position of a cationic feature. The Drude FF predicted a cluster of hydrogen-bond donor features with a more distal cationic feature, whereas the CHARMM pharmacophore predicted fewer hydrogen-bond donors clustered with the cationic feature.

Candidate molecules from SILCS-MC docking were ranked according to their LE scores within each database and FF. The ten highest-ranking compounds from each screening run (or one, in the case of the compound from the Maybridge database using the Drude-derived pharmacophore) are reported in Table S6. Among all screened candidates, CHEMBRIDGE-57665815-3, identified using the Drude-derived pharmacophore, exhibited the most favorable LE. This compound was therefore selected as the primary lead for detailed structural analysis. The molecular structure of CHEMBRIDGE-57665815-3 and its optimized binding pose obtained from SILCS-MC refinement are shown in Figure 10. Visualization of the refined pose within the active site reveals strong correspondence with energetically favorable regions of the FragMaps obtained from the Drude FF. As in the case of the NtMGAM ligand, a cationic heterocycle overlaps with methylammonium nitrogen features. In addition, multiple hydrogen-bond donor and acceptor FragMaps overlaps with complementary functional groups on the ligand (Figure 10).

**Figure 10:**
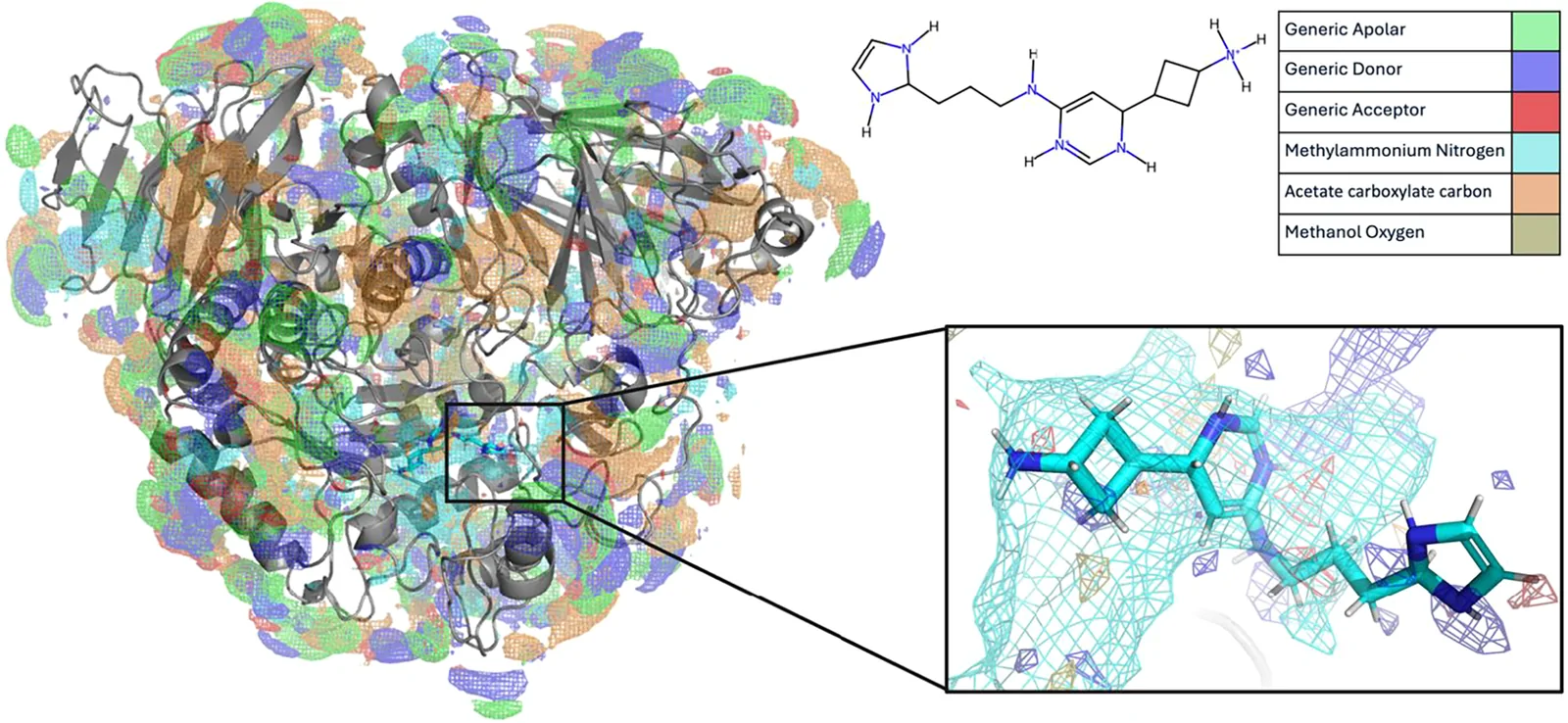
Chemical structure and SILCS-MC-refined binding pose of CHEMBRIDGE-57665815-3 in CtMGAM. The optimized pose is shown within the active site and overlaid with Drude-derived FragMaps.

To further assess the predictive performance of the SILCS-based screening workflow for the C-terminal domain, LGFE and LE scores were calculated for miglitol and acarbose using SILCS-MC pose refinement and compared with the newly identified screening hits (Table S6). Previous experimental studies have established that acarbose is a more effective inhibitor than miglitol for the C-terminal domain. The SILCS-MC predictions obtained using both CHARMM and Drude FFs agree with this trend, with acarbose displaying more favorable LGFE values in both models. Notably, CHEMBRIDGE-57665815-3 exhibits substantially more favorable LGFE and LE values than either of the known inhibitors, suggesting a stronger predicted binding affinity.

### Virtual Screening and Lead Selection for α-Amylase

Application of the CHARMM-derived pharmacophore to the Maybridge database yielded eight candidate compounds, while 100 compounds satisfied the model constraints in the ChemBridge library. In comparison, the Drude-based pharmacophore produced a substantially narrower hit set, identifying one compound from Maybridge and three compounds from ChemBridge. All screened candidates were subsequently evaluated using SILCS-MC docking and the ten highest-ranking compounds from each screening group are summarized in Table S7.

Among all screened molecules, CHEMBRIDGE-34773317-1, identified using the Drude pharmacophore model, exhibited the most favorable LE and was therefore selected as the leading candidate for α-amylase. The chemical structure of CHEMBRIDGE-34773317-1 and its lowest-energy binding pose obtained from SILCS-MC refinement are shown in Figure 11. Examination of the refined pose within the active site demonstrates strong correspondence with the underlying FragMaps. Hydrogen-bond donor and acceptor FragMaps overlap closely with complementary functional groups on the ligand, indicating that this ligand is optimally suited to engage in hydrogen bonding with active-site residues.

**Figure 11:**
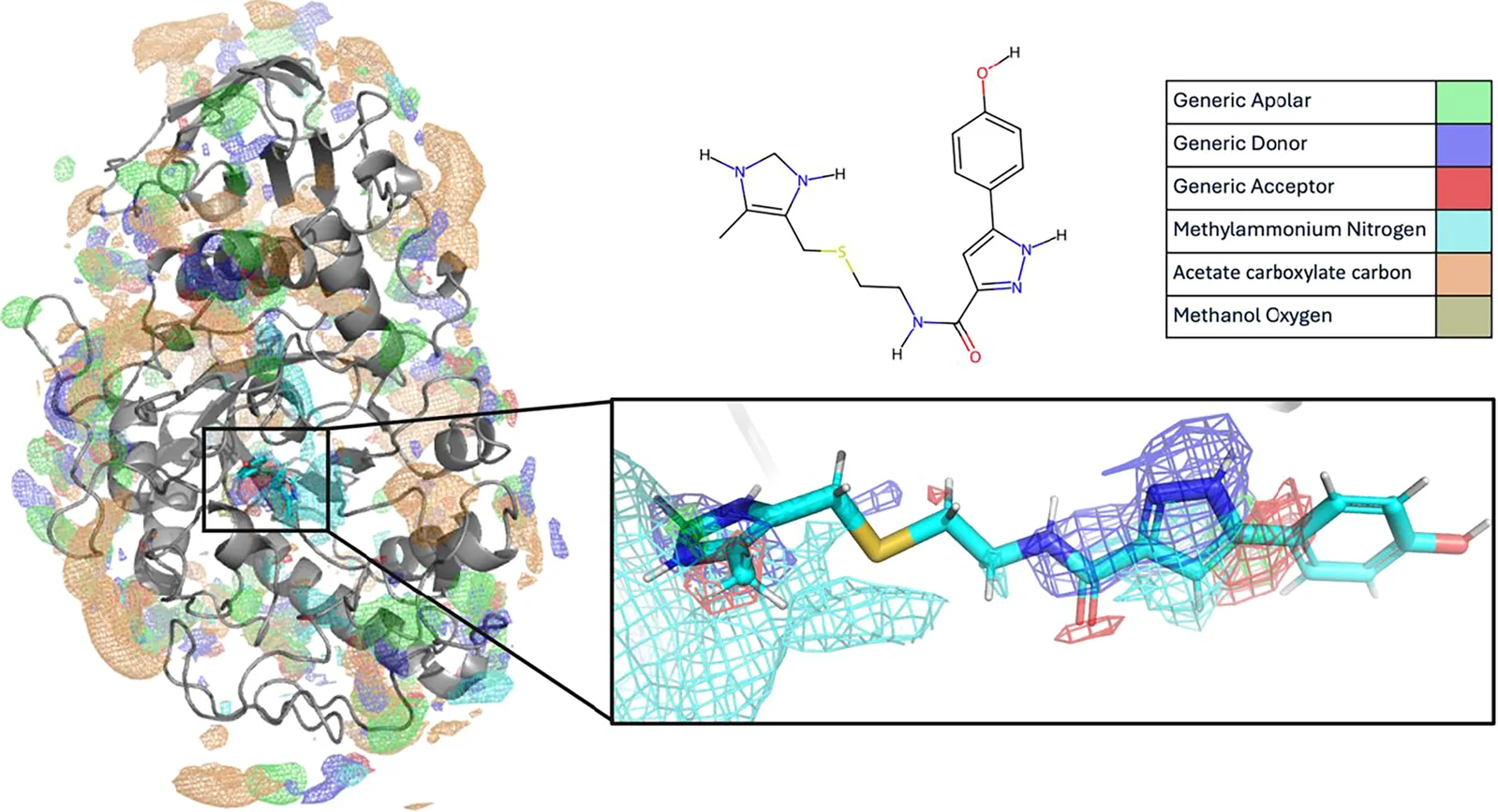
Chemical structure and SILCS-MC-refined binding pose of CHEMBRIDGE-34773317-1 in the α-amylase active site. The optimized pose is shown overlaid with Drude FragMaps.

To further evaluate the predictive performance of the SILCS-based screening workflow, LGFE and LE values calculated for the known inhibitor myricetin were compared with those obtained for CHEMBRIDGE-34773317-1 (Table S7). The newly identified compound had markedly more favorable LGFE and LE scores than myricetin, indicating improved predicted binding affinity and more efficient utilization of molecular interactions within the active site.

Further characterization of the representative hits, including hydrogen-bond occupancies, ligand–protein contact frequencies, and interactions with catalytic residues using the CHARMM additive and Drude polarizable force fields, is presented in our accompanying molecular dynamics study.^54^ Together, the comparison of benchmark inhibitors and newly identified compounds demonstrates that the SILCS–Pharmit–SILCS-MC workflow reproduces established experimental trends across multiple enzyme systems. At the same time, the newly identified lead compounds consistently exhibit superior predicted binding energetics relative to known inhibitors. These findings support the robustness of the screening strategy and highlight the potential of the selected candidates for further optimization and experimental validation.

### Scaffold-Based Clustering of Virtual Screening Hits for NtMGAM

Scaffold-based clustering using the Bemis–Murcko framework indicates that ligands with favorable binding to NtMGAM are characterized by scaffolds that support hydrogen-bond donor and acceptor groups. For the CHARMM-derived hits (Figure 12A,B and Table S8), the most favorable scaffold from the Maybridge dataset is a heterocycle consisting of pyrimidinedione groups, enabling multiple polar interactions within the active site. In addition, the ChemBridge hits are associated with a more compact 2-dihydropyridine scaffold. These trends, as reflected in the LE distributions and representative scaffolds shown in Figure 12 and Table S8, indicate that effective ligands require scaffolds that can engage in polar interactions while maintaining compatibility with hydrophobic regions of the binding pocket.

**Figure 12:**
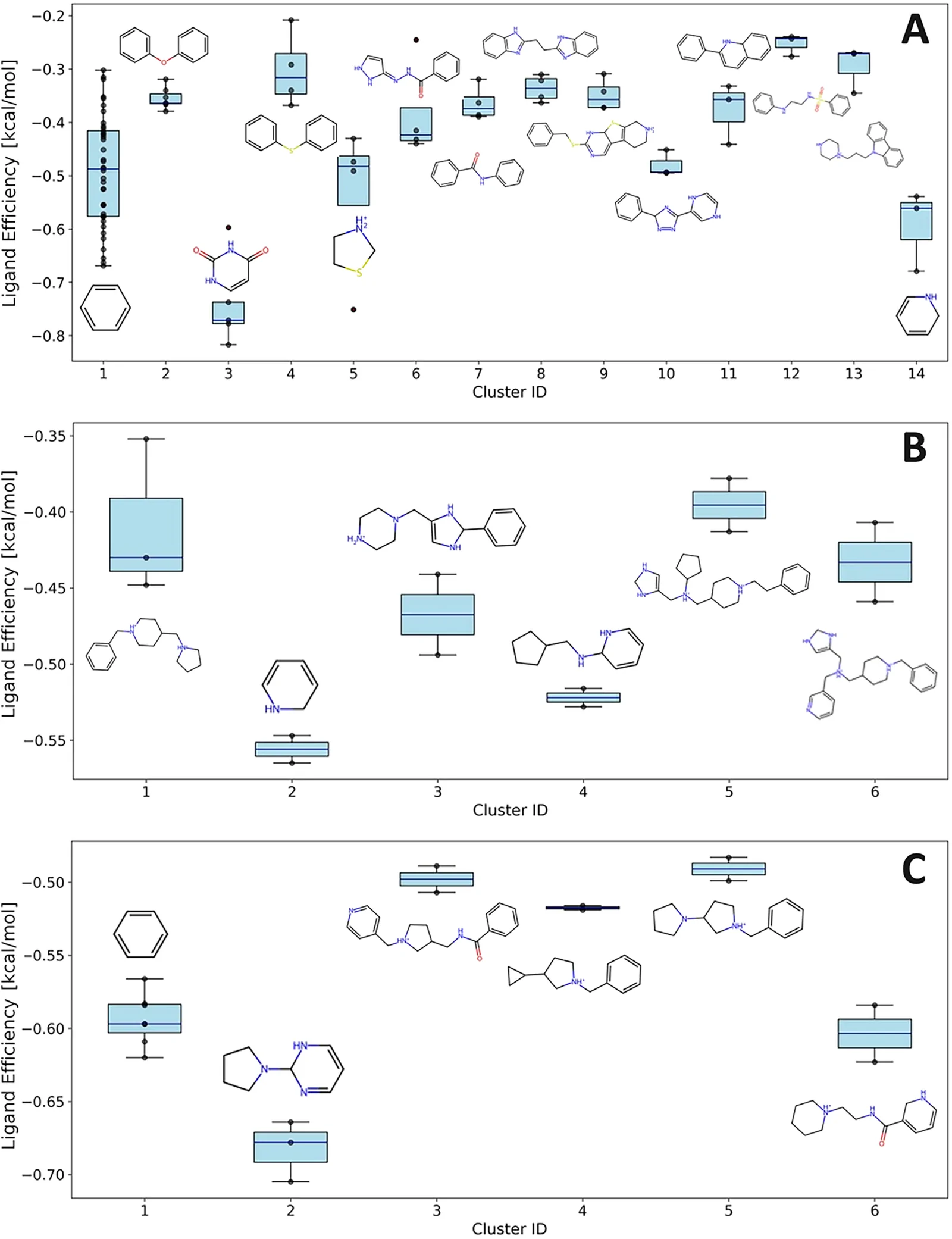
Box plots showing the distribution of LE scores for scaffold-based clusters for NtMGAM. Each box represents the interquartile range with the median indicated by the central line, while individual data points correspond to LE values of compounds within each cluster. The corresponding Bemis–Murcko scaffold structures for each cluster are displayed alongside the plots. Results are shown for (A) CHARMM FF screening against the Maybridge database, (B) CHARMM FF screening against the ChemBridge database, and (C) Drude polarizable FF screening against the ChemBridge database.

For the Drude model, the limited number of Maybridge hits prevented clustering; however, clustering of the ChemBridge hits (Figure 12C and Table S8) reveals a consistent preference for scaffolds composed of linked heteroaromatic rings enriched in hydrogen-bond donor and acceptor sites. This trend suggests that the use of a polarizable FF leads to ligands that feature aromatic frameworks with multiple heteroatoms that are better able to engage the highly charged active site through directional hydrogen bonding and enhanced electrostatic interactions.

Together, CHARMM and Drude clustering results are consistent with the FragMaps and pharmacophore features generated for this enzyme, which highlight dominant hydrogen-bond donor, acceptor, and polar interaction regions. Optimal ligands for NtMGAM should incorporate heteroaromatic ring structures with polar functionalities that align with the interaction hotspots.

### Scaffold-Based Clustering of Virtual Screening Hits for CtMGAM

Scaffold-based clustering using the Bemis–Murcko framework for CtMGAM indicates that favorable ligands are characterized by heterocyclic scaffolds that combine polar and aromatic functionalities (Figure 13A,B and Table S9). For the Maybridge dataset (Figure 13A), three clusters were identified, with the most favorable cluster characterized by sulfur- and nitrogen-containing heterocycles, including protonated amine groups, suggesting a strong contribution from electrostatic interactions and hydrogen bonding. In addition, the ChemBridge dataset yielded two clusters (Figure 13B), with the more favorable cluster dominated by polyaromatic scaffolds containing amide functionalities, indicating that effective ligands can also utilize a combination of π-interactions and hydrogen-bonding groups. These results from CHARMM workflow suggest that ligands must balance aromatic interactions with appropriately positioned polar groups to interact with the binding pocket. This outcome is consistent with the FragMaps and pharmacophore features generated for CtMGAM, which highlight contributions from hydrogen-bond donor, acceptor, and apolar interaction regions. Together, these results suggest that optimal ligands for CtMGAM should incorporate heterocyclic or polyaromatic frameworks with strategically placed polar functionalities capable of engaging both electrostatic and hydrophobic interaction sites. Using the Drude FF, the limited number of hits prevented meaningful Bemis–Murcko clustering, indicating reduced sampling of compatible scaffolds for this system.

**Figure 13:**
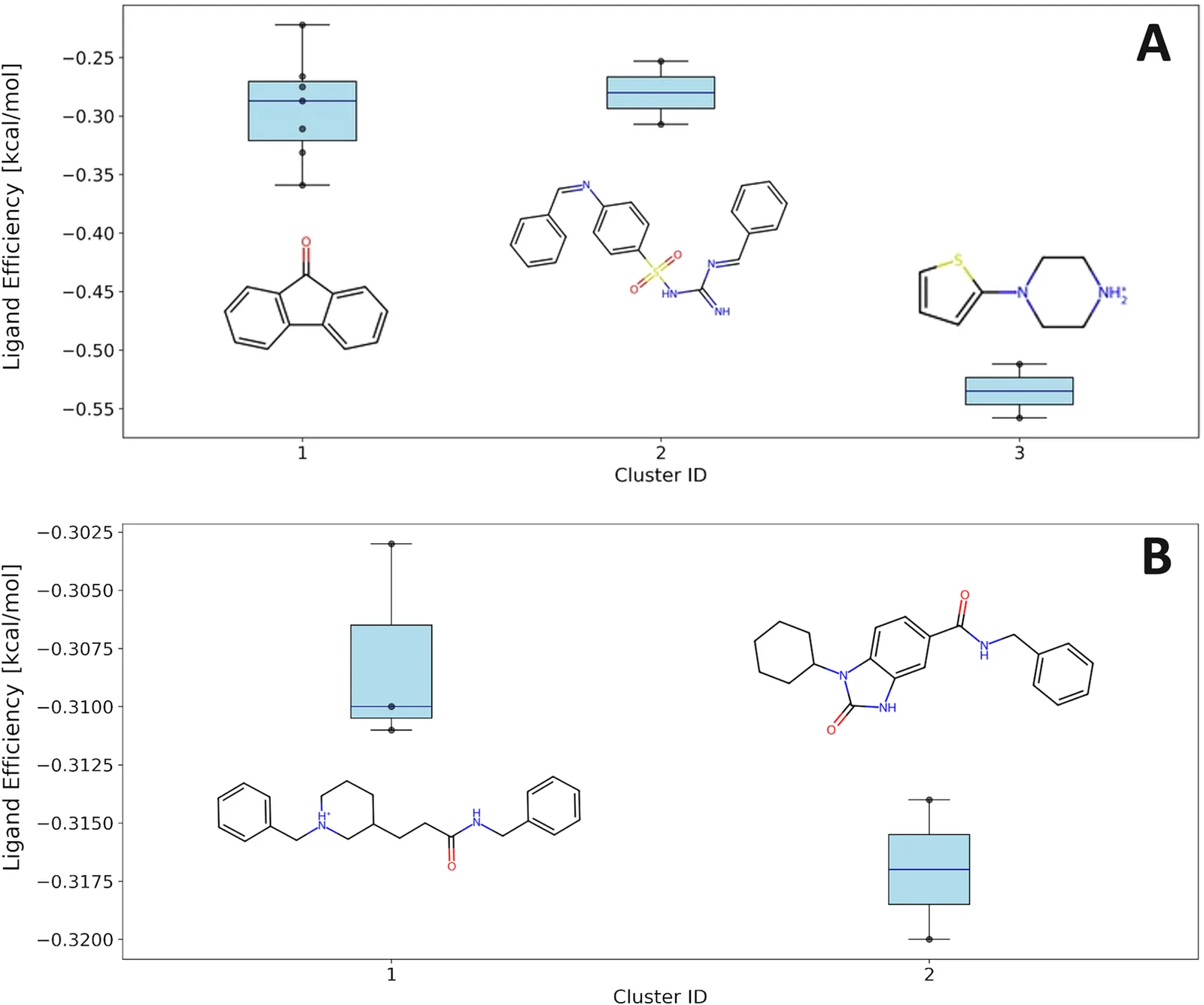
Box plots showing the distribution of LE scores for scaffold-based clusters for CtMGAM. Each box represents the interquartile range with the median indicated by the central line, while individual data points correspond to LE values of compounds within each cluster. The corresponding Bemis–Murcko scaffold structures for each cluster are displayed alongside the plots. Results are shown for (A) CHARMM FF screening against the Maybridge database and (B) CHARMM FF screening against the ChemBridge database.

### Scaffold-Based Clustering of Virtual Screening Hits for α-Amylase

Scaffold-based clustering using the Bemis–Murcko framework was performed for α-amylase (Table S10). From the Maybridge dataset, a single cluster containing two compounds was obtained, featuring a simple aromatic scaffold, indicating that aromatic moieties can support binding when appropriately positioned. In the ChemBridge dataset, one larger cluster was identified, characterized by a scaffold that combines piperidine rings linked to an aromatic ring via an amide. This arrangement suggests that effective ligands for α-amylase require a combination of aromatic character with polar and positively charged functionalities to enable simultaneous hydrophobic, electrostatic, and hydrogen-bonding interactions within the active site.

In contrast, application of the Drude FF resulted in too few hits to allow for Bemis–Murcko clustering, indicating limited sampling of compatible scaffolds using the polarizable model. Despite this outcome, the structural trends observed from the CHARMM-based clustering align with the FragMaps and pharmacophore features generated for α-amylase, which emphasize the importance of hydrogen-bond donor, acceptor, and charged interaction regions. Together, these results suggest that optimal ligands for α-amylase should incorporate aromatic scaffolds functionalized with polar and cationic groups that match the electrostatic and hydrogen-bonding hotspots of the binding site.

### Fingerprint-Based Clustering of Virtual Screening Hits for NtMGAM

Fingerprint-based clustering of NtMGAM hits using Morgan fingerprints and agglomerative clustering (Supporting Information, Figures S2–S5) shows that favorable ligands group into chemically distinct series defined primarily by their functional group composition. For the CHARMM-derived hits (Supporting Information, Figures S2 and S3), the most favorable chemotypes correspond to Cluster 4 (Maybridge) and Cluster 2 (ChemBridge), which are enriched in aromatic scaffolds combined with strong hydrogen-bonding functionalities. In particular, the Maybridge cluster is associated with nitro-, aromatic, amide-containing functional groups, while the ChemBridge cluster favors amide/amine-rich chemotypes with reduced aromatic character. These results indicate that different structural classes can achieve favorable binding, but all rely on appropriately positioned polar groups. The Drude-derived hits showed a similar preference for aromatic structures containing polar functional groups, with detailed clustering results provided in the Supporting Information (Figures S4 and S5).

### Fingerprint-Based Clustering of Virtual Screening Hits for CtMGAM

For the CHARMM-derived CtMGAM hits, fingerprint-based clustering identified distinct chemotypes in both the Maybridge and ChemBridge datasets, with the more favorable clusters generally enriched in polar functional groups capable of hydrogen-bonding and electrostatic interactions (Figures S6 and S7). Detailed cluster compositions are provided in the Supporting Information.

Using the Drude FF, clustering was only possible for the ChemBridge dataset, for which two clusters were identified (Supporting Information, Figures S8 and S9). The most favorable cluster (Cluster 2) is associated with alkene and non-aromatic, nitrogen-rich frameworks containing amine, imine, and hydroxyl groups, indicating that polar functionalities are particularly effective when considering explicit electronic polarization. In contrast, the less favorable cluster is dominated by aromatic and amide-containing compounds, suggesting a reduced contribution from rigid aromatic interactions. This shift relative to the CHARMM results indicates that incorporation of polarization enhances the importance of electrostatic complementarity. These results suggest that ligands targeting CtMGAM may benefit from prioritizing flexible scaffolds with multiple polar functional groups positioned to engage the interaction hotspots identified in the FragMaps and pharmacophore models. This agreement further supports that the dominant interactions captured in the FragMaps are effectively reflected in the ligand chemotypes identified through clustering.

### Fingerprint-Based Clustering of Virtual Screening Hits for α-Amylase

Fingerprint-based clustering of the α-amylase hits obtained with the CHARMM FF (Supporting Information, Figures S10 and S11) shows that favorable compounds are distributed across distinct chemotypes. For the Maybridge dataset, three clusters were identified, with Cluster 3 showing the most favorable LE despite its small size. This cluster combines aromatic ring systems with amine, ester, ether, and hydroxyl functionalities, indicating that a balance of aromatic character and multiple hydrogen-bonding groups contributes to improved binding. In contrast, Cluster 1 represents the most populated chemotype and is dominated by amide- and aromatic-containing compounds, suggesting that these features are common but not necessarily optimal.

For the ChemBridge dataset, favorable clusters similarly contained combinations of polar and aromatic or unsaturated functionalities, with detailed cluster compositions provided in the Supporting Information (Figures S10 and S11). In contrast, the Drude FF yielded too few hits from either database to support meaningful fingerprint-based clustering. Overall, the CHARMM-based clustering trends are consistent with the SILCS FragMaps and pharmacophore features for α-amylase, which emphasize hydrogen-bond donor, acceptor, and polar interaction regions within the active site. Together, these results indicate that favorable α-amylase ligands should incorporate aromatic or partially unsaturated scaffolds with appropriately positioned polar functional groups (e.g., hydroxyl, amine, amide, and ether functionalities) to effectively engage these interaction hotspots.

## Conclusions

In this study, we developed an enhanced SILCS-based computational framework and applied it to three enzymes implicated in type 2 diabetes: the N-terminal and C-terminal domains of maltase–glucoamylase and α-amylase. We systematically evaluated the impact of fragment-sampling strategy and force field choice on binding-site characterization, pharmacophore development, and virtual screening performance. Across all systems, outcomes from the conventional mixed-probe SILCS workflow were dominated by cationic fragments, particularly in highly charged active sites. This dominance suppressed sampling of hydrogen-bond donor, acceptor, and hydrophobic interaction sites, limiting chemical diversity in the resulting FragMaps. The neutral–charged SILCS protocol consistently alleviated this limitation by separating neutral and charged probe molecules, thereby introducing other chemically relevant interaction features. This improvement was observed for all three enzymes.

Comparing the CHARMM and Drude simulations showed that explicit electronic polarization further refines the binding-site representation. While separation of probes was the primary driver of improved sampling, application of the Drude polarizable FF enhanced resolution of electrostatic and hydrogen-bonding hotspots and produced more energetically diverse pharmacophore features. However, the Drude workflow implemented here was initialized from GCMC-sampled configurations generated by prior CHARMM additive SILCS simulations, which should be considered when selecting between the additive and polarizable approaches. Pharmacophore models derived from neutral–charged FragMaps successfully captured dominant interaction motifs within each binding site. Subsequent virtual screening, SILCS-MC refinement, and ligand efficiency analysis enabled prioritization of candidate inhibitors. For NtMGAM, both the CHARMM and Drude FFs produced similar results but the FragMaps from the Drude FF led to the best predicted inhibitors for CtMGAM and α-amylase. Integration of Bemis–Murcko and fingerprint-based clustering revealed structural motifs and core frameworks associated with favorable binding energetics, providing insight into structure–activity relationships.

In conclusion, for the three highly charged enzyme systems studied here, the neutral–charged SILCS approach reduced the dominance of charged-probe sampling and increased the representation of donor, acceptor, and apolar interaction features within the active sites. The conventional workflow remains suitable when it produces converged FragMaps with a balanced representation of relevant interaction types, whereas the neutral–charged protocol is recommended for highly charged or strongly polar binding sites when charged probes suppress sampling of donor, acceptor, or apolar features. The neutral–charged protocol should therefore be viewed as a targeted strategy for mitigating competitive exclusion rather than as a universal replacement for the conventional workflow. The resulting workflow supports pharmacophore modeling, virtual screening, and candidate inhibitor prioritization for the highly charged enzymatic systems investigated here in the context of type 2 diabetes.

## Supplementary Material



## Data Availability

Pharmacophore files and relevant small-molecule and protein coordinates are available via Zenodo at 10.5281/zenodo.20648126.

## Notes

Supporting methods for conventional CHARMM–SILCS GCMC setup, SILCS-MC scoring and atom classification, and Pharmit virtual screening; additional fingerprint-based clustering results for NtMGAM, CtMGAM, and α-amylase; atom-wise GFE analysis of SILCS-MC-refined poses; UMAP clustering and functional-group heatmaps; FragMap definitions and overlap coefficients; energetic and spatial properties of pharmacophore features; top-ranked virtual screening compounds and SILCS-MC scoring results; representative scaffold structures and statistical parameters from Bemis–Murcko clustering (PDF)
