# Supplementary material for: Computer-Aided Drug Design for Type 2 Diabetes-Related Enzymes: SILCS-Based Fragment Mapping, Pharmacophore Modeling, and Virtual Screening
Source: J Chem Inf Model. 2026 Sep 30;66(19):12969–88. doi: 10.1021/acs.jcim.6c01733 (PMC13663488; doi:10.1021/acs.jcim.6c01733)
Supplement: Supplementary file 1 [file ci6c01733_si_001.pdf]

SUPPORTING INFORMATION

Computer-Aided Drug Design for Type 2 Diabetes-Related Enzymes: SILCS-Based Fragment Mapping, Pharmacophore Modeling, and Virtual Screening

Esmat Mohammadi[1,2] and Justin A. Lemkul[2,3,*]

[1] Department of Chemical Engineering, Virginia Tech, Blacksburg, VA 24061, USA
[2] Department of Biochemistry, Virginia Tech, Blacksburg, VA 24061, USA
[3] Center for Computer-Aided Drug Design, Virginia Tech, Blacksburg, VA 24061, USA

SUPPLEMENTARY METHODS

Conventional CHARMM–SILCS GCMC Setup

After system setup, each protein system was subjected to grand canonical Monte Carlo (GCMC) sampling. A GCMC-active region was defined around the protein, extending 15 Å beyond the protein in all directions. All water and solute molecules within this region were removed before refilling.[1] Solute and solvent refilling was performed in two stages. In the first stage, all fragment probe molecules were inserted into the GCMC region until their target concentration of approximately 0.25 M was reached.[1, 2] The probe molecules were sampled simultaneously and competed with one another for favorable interaction sites around the protein. In the second stage, water molecules were added to restore the solvent concentration to approximately 55 M.[1] Each refilling stage consisted of 200,000 GCMC steps, during which insertion, deletion, translation, and rotation moves were attempted and accepted or rejected based on the Metropolis criterion using the excess chemical potential of each species.[3]

SILCS-MC Scoring and Atom Classification

The SILCS Monte Carlo (SILCS-MC) method was used to predict ligand binding poses and estimate binding affinities using the SILCS FragMaps generated from the CHARMM or Drude simulations.[1] This approach employs MC sampling to explore ligand translational, rotational, and conformational degrees of freedom within the protein binding site, while energetically evaluating interactions based on the underlying FragMaps.[4]

Within the SILCS-MC framework, ligand atoms were assigned to specific atom types using the CHARMM General Force Field (CGenFF)[5] enabling mapping onto the SILCS atom classification scheme and direct correspondence with the appropriate FragMaps. During MC sampling, ligand conformations were evaluated based on how favorably their atom types

overlapped with energetically preferred regions of the FragMaps, and ligand grid free energy (LGFE) scores were calculated by summing the grid free energy contributions from all ligand atoms.[6] The lowest LGFE from clustered poses were used to rank compounds and identify favorable binding modes.

Pharmit Virtual Screening

Pharmacophore features derived from SILCS-Pharm served as the primary queries for database searching. Candidate molecules were evaluated based on spatially matching these features within the binding site. Pharmit employs the Pharmer algorithm to establish geometric relationships among pharmacophore features by constructing triangular patterns defined by precise three-dimensional coordinates.[45] This approach enables accurate alignment of library compounds to the query model and allows quantitative assessment of binding compatibility through root-mean-square deviation (RMSD) calculations.

SUPPLEMENTARY RESULTS

Additional Fingerprint-Based Clustering Results

NtMGAM

Using the Drude polarizable model, fewer clusters were identified, with Cluster 3 (Maybridge) and Cluster 2 (ChemBridge) showing the most favorable LE (Supporting Information, Figures S3 and S4). These clusters are characterized by aromatic systems combined with polar functionalities, particularly hydroxyl groups and other oxygen- and nitrogen-containing groups such as amines, amides, and ethers, indicating a stronger dependence on electrostatic and hydrogen-bonding interactions. These trends are consistent with the scaffold-based analysis and

the SILCS-derived FragMaps and pharmacophore features, which emphasize hydrogen-bond donor, acceptor, and polar interaction regions. Together, these results suggest that optimal ligands for NtMGAM should incorporate polar functional groups into the scaffold to match these interaction hotspots.

CtMGAM

Fingerprint-based clustering of CtMGAM hits resulted from CHARMM workflow (Supporting Information, Figures S5 and S6) reveals that LE is associated with distinct functional group combinations rather than a single dominant scaffold type. Visualization of the clustering results in UMAP space (Supporting Information, Figure S5A,B), together with LE distributions (Supporting Information, Figure S5C,D) and functional group heatmaps (Supporting Information, Figure S6), shows that the most favorable compounds are grouped into chemically coherent series. In the Maybridge dataset, Cluster 5 corresponds to the most favorable chemotype and is defined by compounds containing amine, amide, hydroxyl, and ether functionalities, with comparatively low aromatic and nitro content. In contrast, clusters dominated by aromatic, nitro-containing scaffolds (e.g., Clusters 1 and 2) are associated with reduced binding efficiency, suggesting that rigid, aromatic frameworks are not as well-suited for this binding site.

A similar pattern was observed for the ChemBridge dataset, for which Cluster 9 shows the most favorable binding behavior. This cluster is characterized by nitrogen-containing functional groups, including amines and imines, together with hydroxyl functionalities and moderate aromatic character. Despite differences in chemotype composition between the two libraries, both datasets highlight the importance of polar functional groups capable of hydrogen bonding and electrostatic interactions. This behavior is consistent with the SILCS FragMaps and pharmacophore features for CtMGAM, which highlight key donor, acceptor, and apolar interaction regions within the binding pocket.

α-Amylase

For the ChemBridge dataset, Cluster 33 exhibits the most favorable LE but is limited in size, whereas Cluster 15 provides a more representative chemotype among the larger groups.

Cluster 33 is defined by strong hydrogen-bonding character, with consistent hydroxyl and amide functionalities and additional contributions from amine, aromatic, and sulfur-containing groups, indicating a compact polar interaction pattern. In contrast, Cluster 15 reflects a more diverse and flexible chemotype, combining amine-rich scaffolds with imine and alkene functionalities, together with moderate aromatic and amide content. These differences suggest that both compact polar frameworks and more flexible, unsaturated nitrogen-containing scaffolds can support binding, although through distinct interaction strategies.

SUPPORTING FIGURES

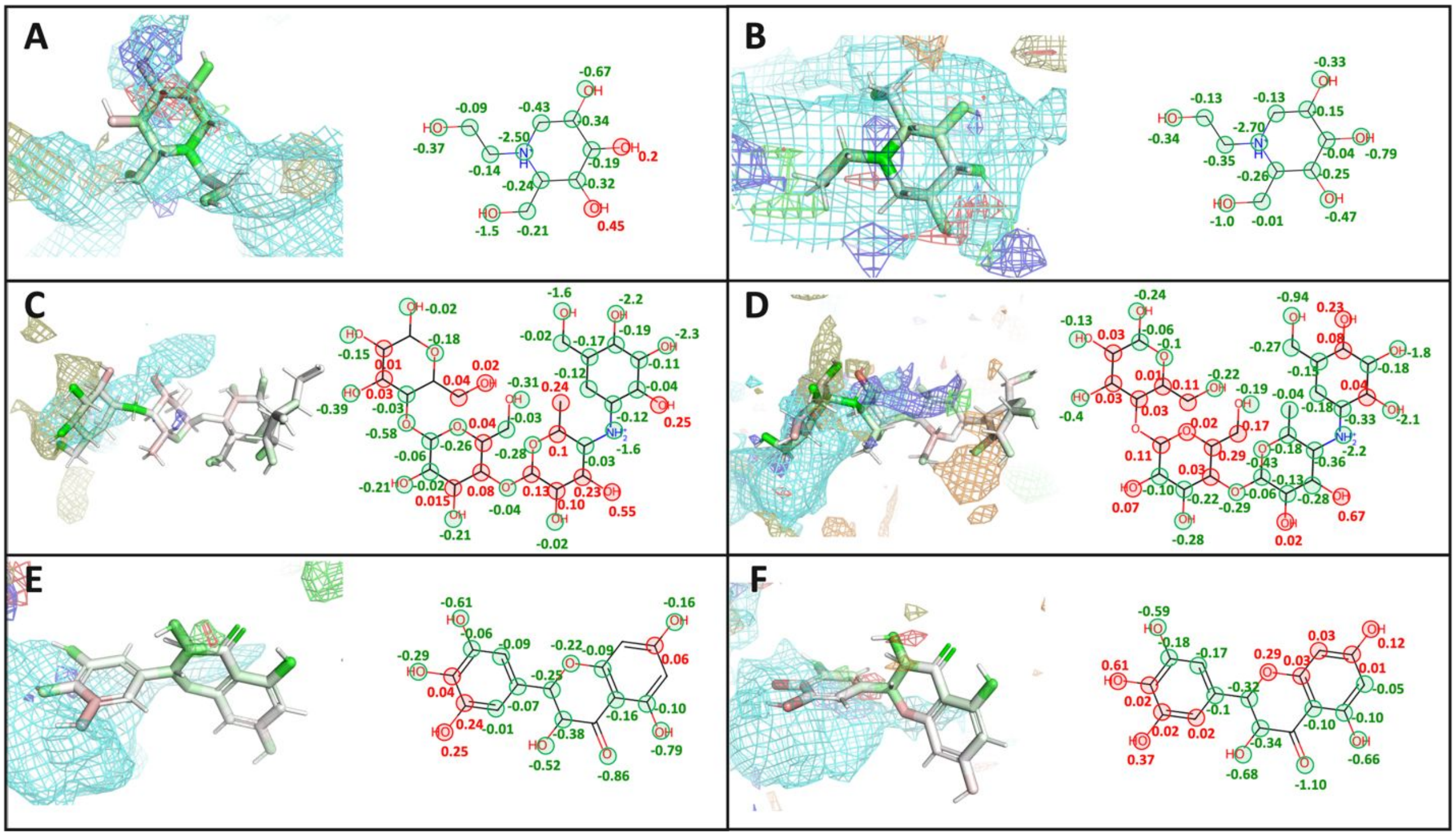

Figure S1. Atom-wise GFE analysis of the SILCS-MC-refined poses of known inhibitors using the CHARMM and Drude FragMaps: miglitol in NtMGAM using (A) CHARMM and (B) Drude, acarbose in CtMGAM using (C) CHARMM and (D) Drude, and myricetin in α-amylase using (E) CHARMM and (F) Drude. Ligand atoms are colored from green to red according to their GFE contributions, with green indicating favorable and red indicating unfavorable contributions. The corresponding atom-wise GFE values, in kcal/mol, are shown in the 2D ligand representations.

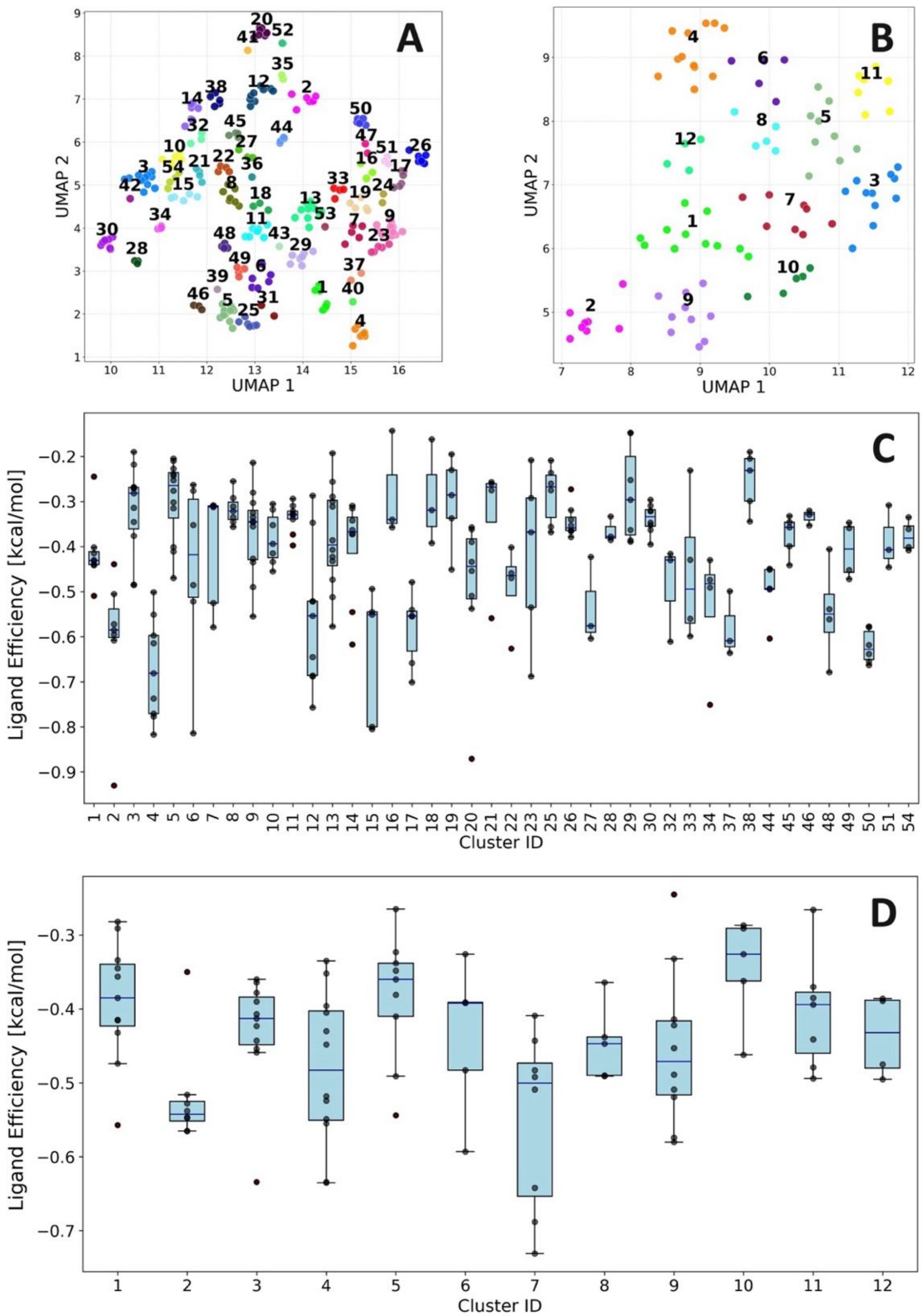

Figure S2. Two-dimensional UMAP projection of ligand fingerprint–based clustering of compounds identified for NtMGAM using the CHARMM FF. Compounds were obtained from (A) the Maybridge database and (B) the ChemBridge database and grouped based on structural similarity. Each point represents a ligand, and colors indicate cluster membership. Box plots of LE scores for clusters containing at least three compounds identified from (C) Maybridge and (D) ChemBridge databases.

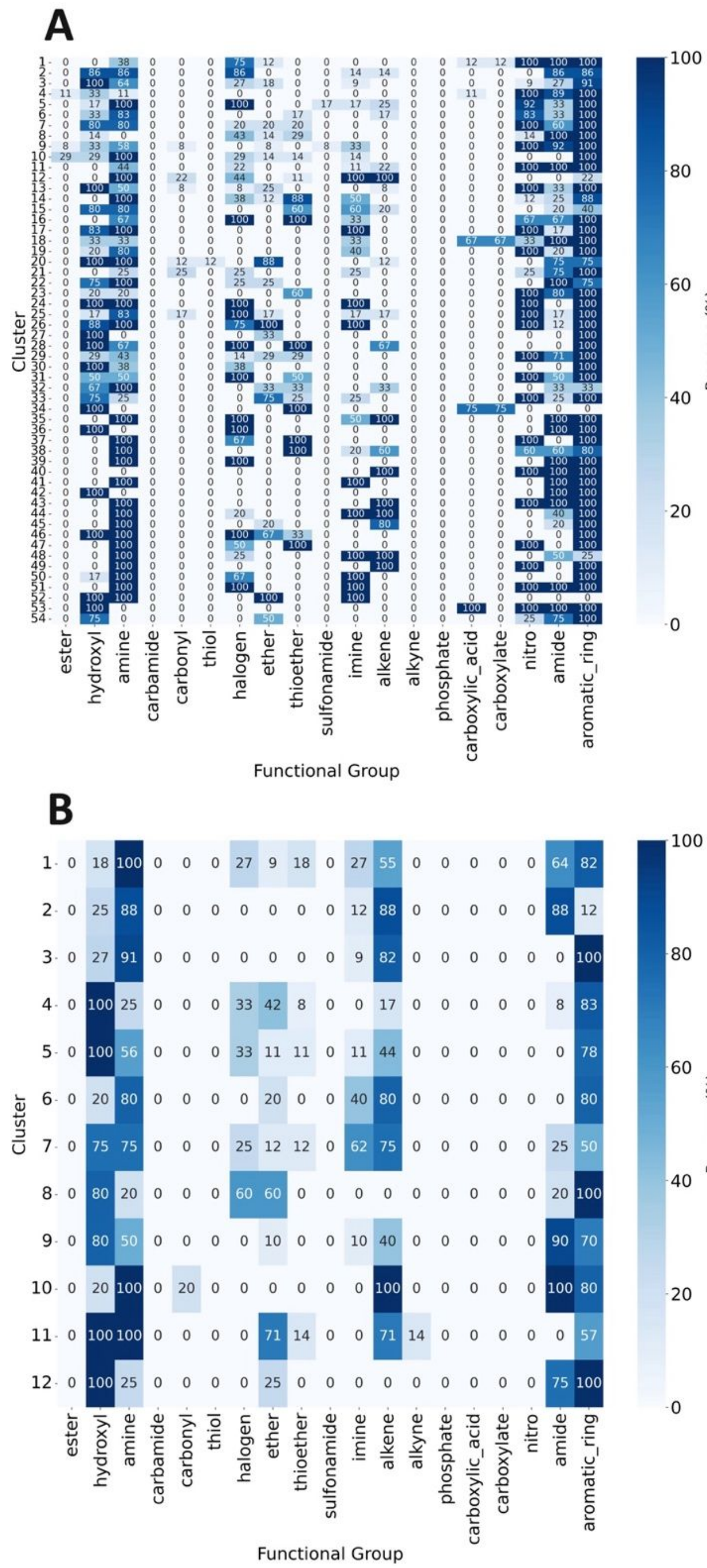

Figure S3. Heatmap showing the percentage occurrence of major functional groups within each cluster identified for NtMGAM using the CHARMM FF. Clusters were derived from compounds obtained from (A) the Maybridge database and (B) the ChemBridge database. Values represent the proportion of ligands in each cluster containing the indicated functional group.

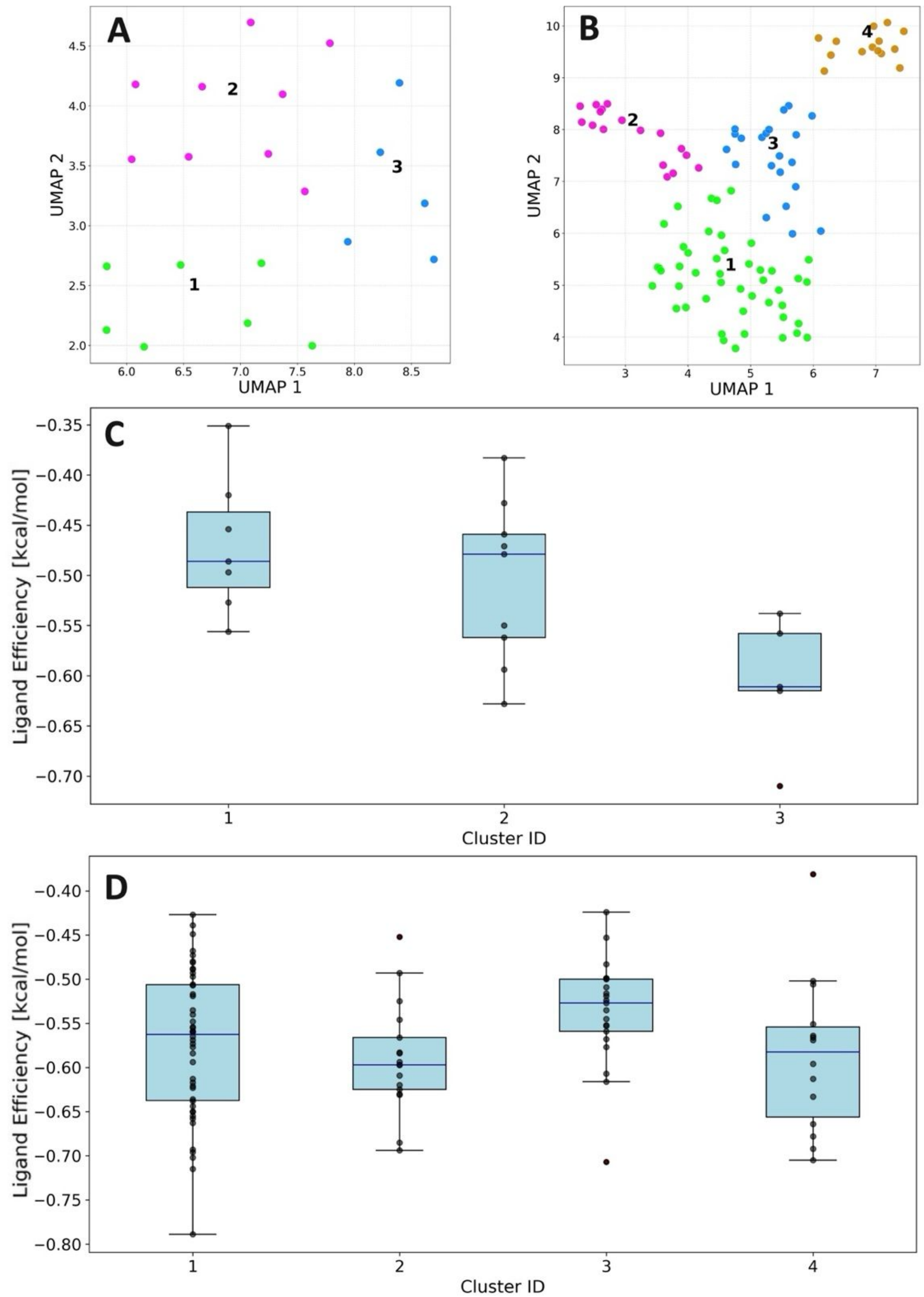

Figure S4. Two-dimensional UMAP projection of ligand fingerprint–based clustering of compounds identified for NtMGAM using the Drude FF. Compounds were obtained from (A) the Maybridge database and (B) the ChemBridge database and grouped based on structural similarity. Each point represents a ligand, and colors indicate cluster membership. Box plots of LE scores for clusters containing at least three compounds identified from (C) Maybridge and (D) ChemBridge databases.

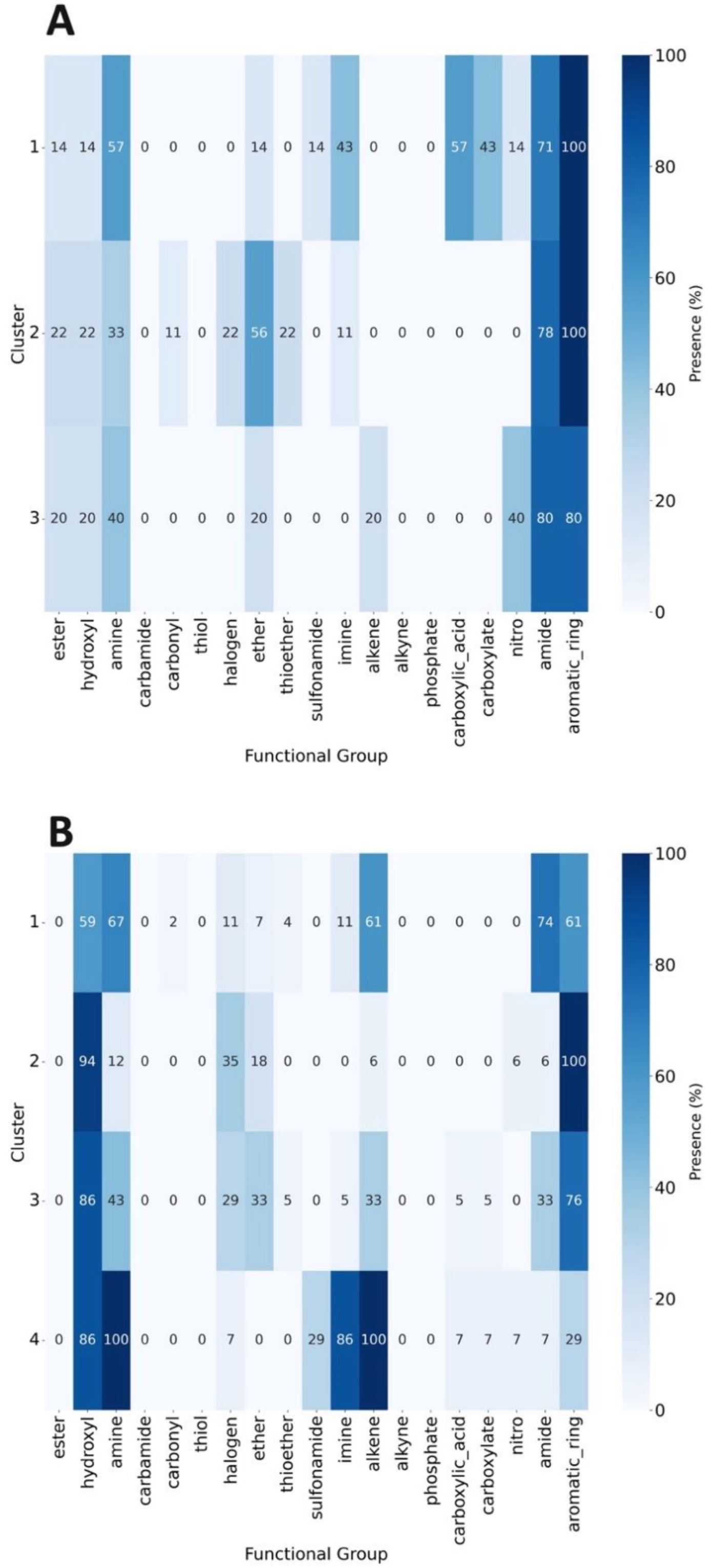

Figure S5. Heatmap showing the percentage occurrence of major functional groups within each cluster identified for NtMGAM using the Drude FF. Clusters were derived from compounds obtained from (A) the Maybridge database and (B) the ChemBridge database. Values represent the proportion of ligands in each cluster containing the indicated functional group.

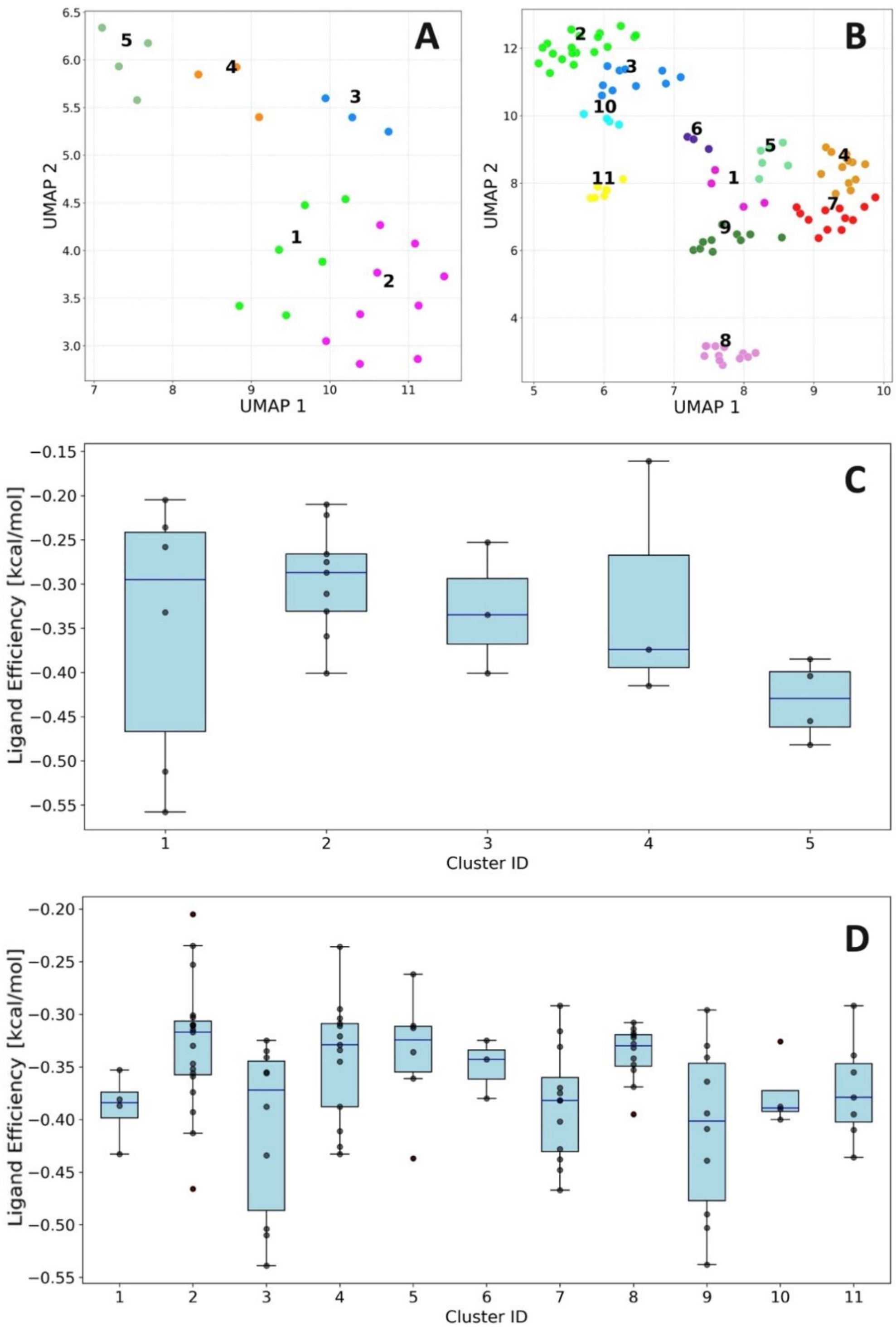

Figure S6. Two-dimensional UMAP projection of ligand fingerprint–based clustering of compounds identified for CtMGAM using the CHARMM FF. Compounds were obtained from (A) the Maybridge database and (B) the ChemBridge database and grouped based on structural similarity. Each point represents a ligand, and colors indicate cluster membership. Box plots of LE scores for clusters containing at least three compounds identified from (C) Maybridge and (D) ChemBridge databases.

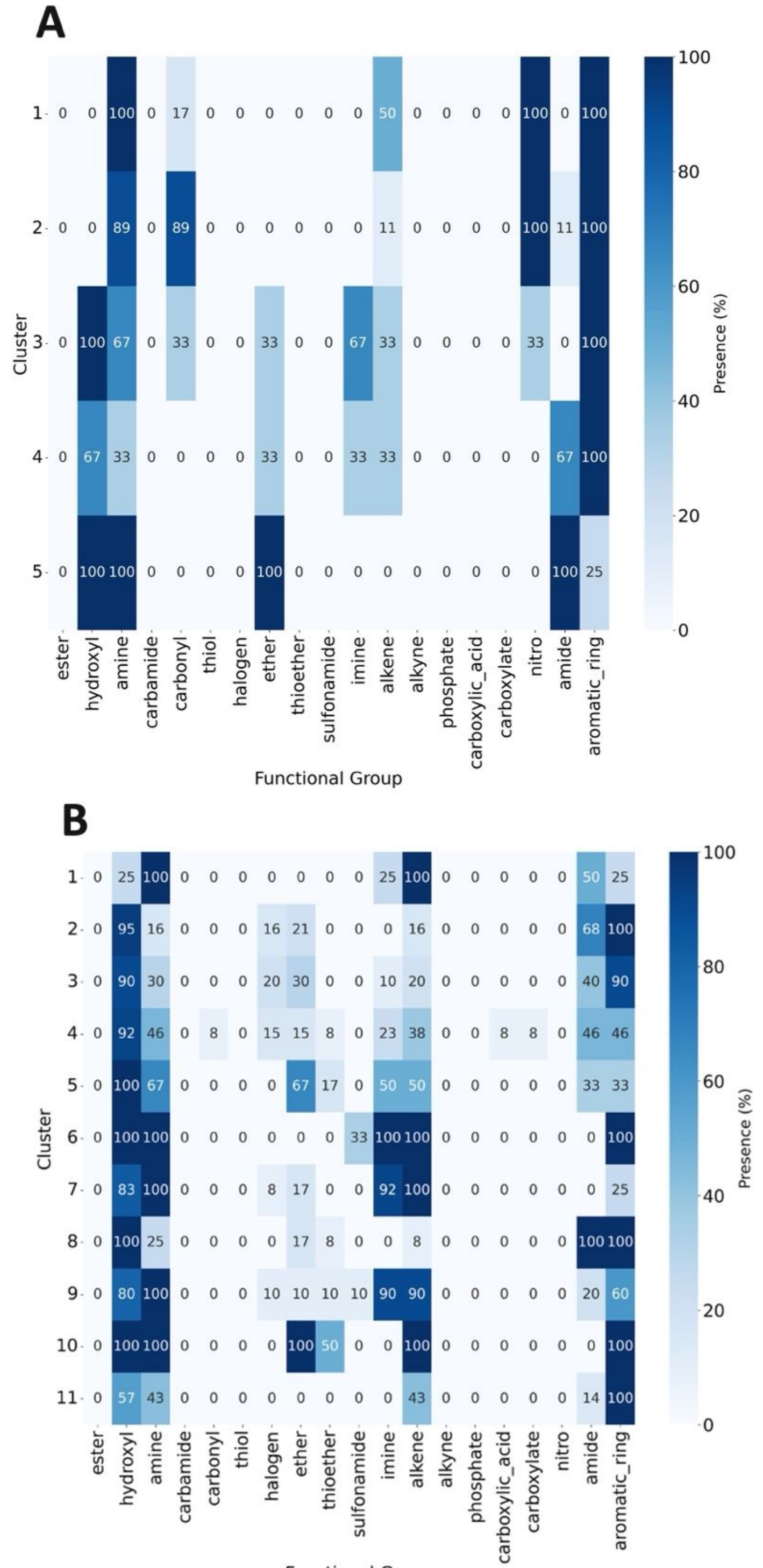

Figure S7. Heatmap showing the percentage occurrence of major functional groups within each cluster identified for CtMGAM using the CHARMM FF. Clusters were derived from compounds obtained from (A) the Maybridge database and (B) the ChemBridge database. Values represent the proportion of ligands in each cluster containing the indicated functional group.

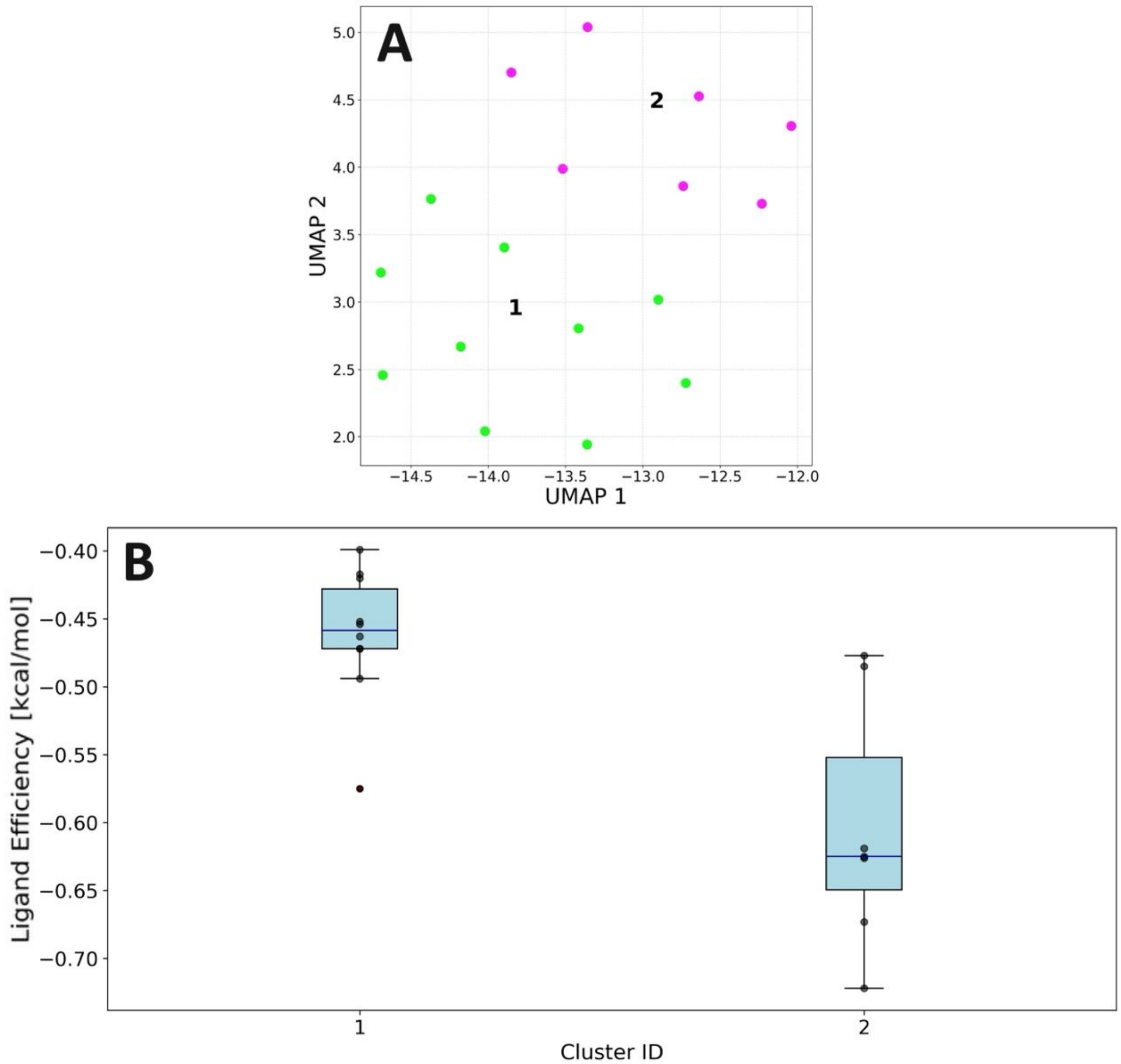

Figure S8. (A)Two-dimensional UMAP projection of ligand fingerprint–based clustering of compounds identified for CtMGAM using the Drude FF. Compounds were obtained from ChemBridge database and grouped based on structural similarity. Each point represents a ligand, and colors indicate cluster membership. (B) Box plots of ligand efficiency (LE) scores for clusters identified from ChemBridge databases.

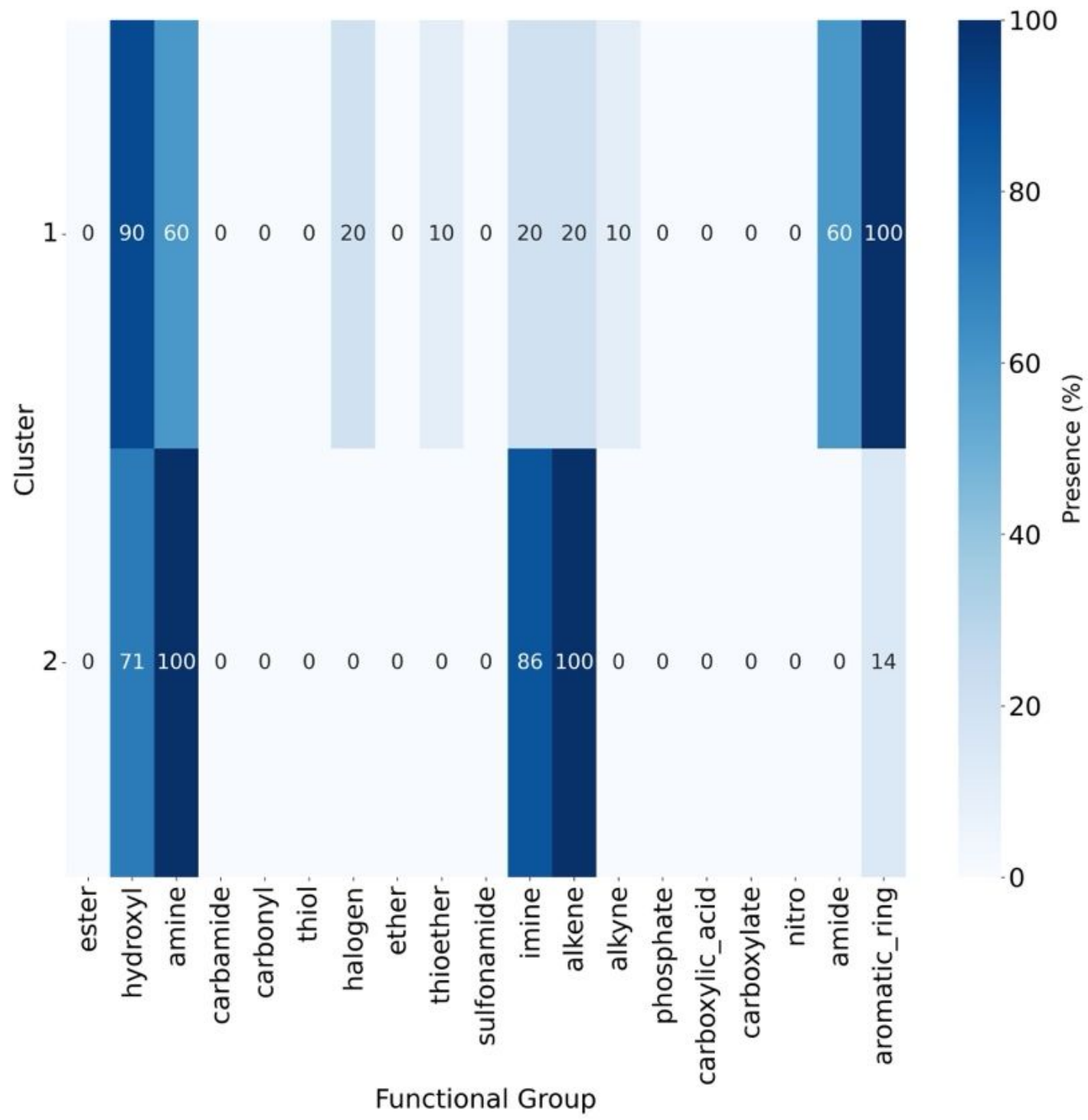

Figure S9. Heatmap showing the percentage occurrence of major functional groups within each cluster identified for CtMGAM using the Drude FF. Clusters were derived from compounds obtained from ChemBridge database. Values represent the proportion of ligands in each cluster containing the indicated functional group.

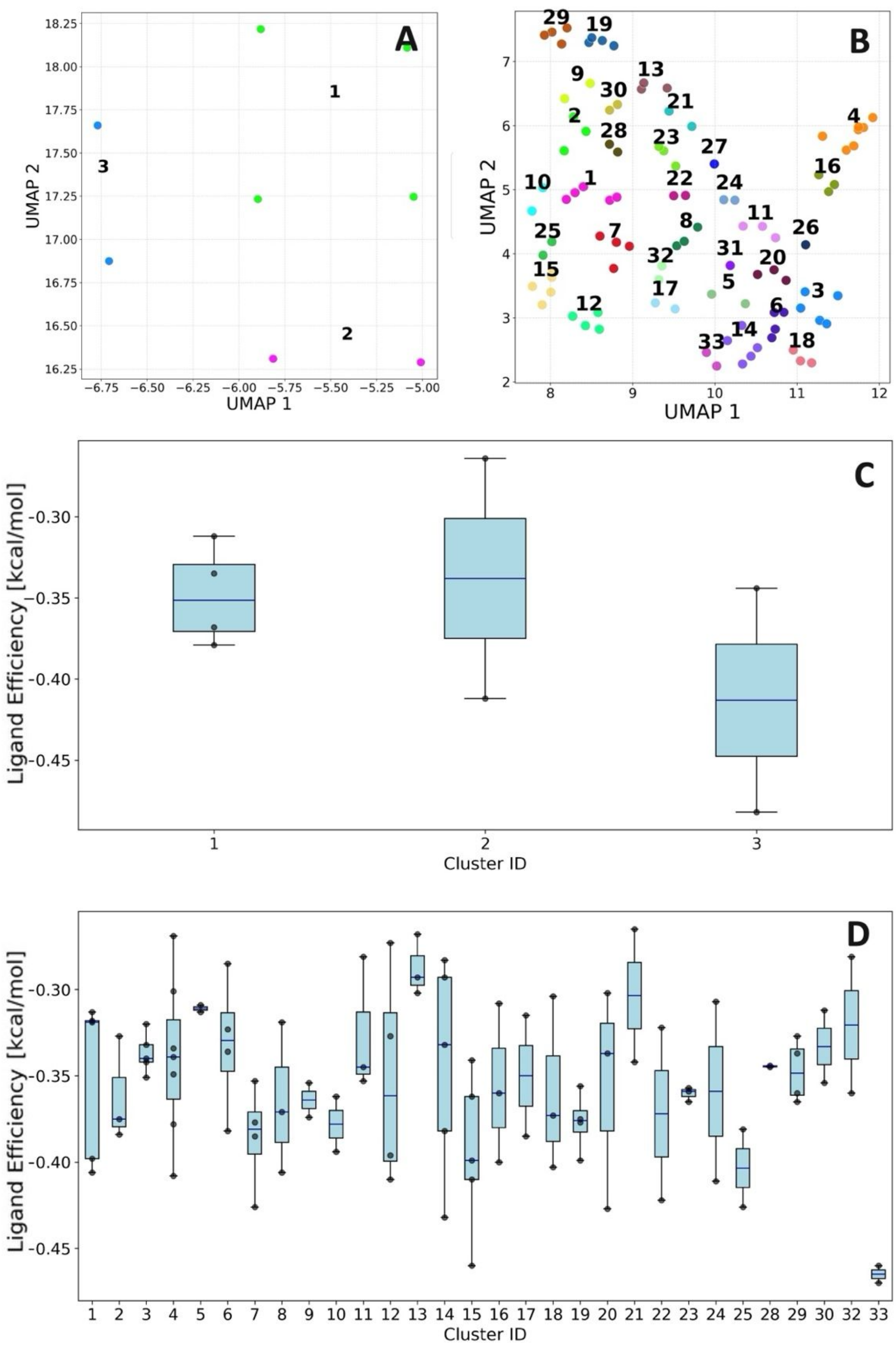

Figure S10. Two-dimensional UMAP projection of ligand fingerprint–based clustering of compounds identified for α-amylase using the CHARMM FF. Compounds were obtained from (A) the Maybridge database and (B) the ChemBridge database and grouped based on structural similarity. Each point represents a ligand, and colors indicate cluster membership. Box plots of LE scores for clusters containing at least three compounds identified from (C) Maybridge and (D) ChemBridge databases.

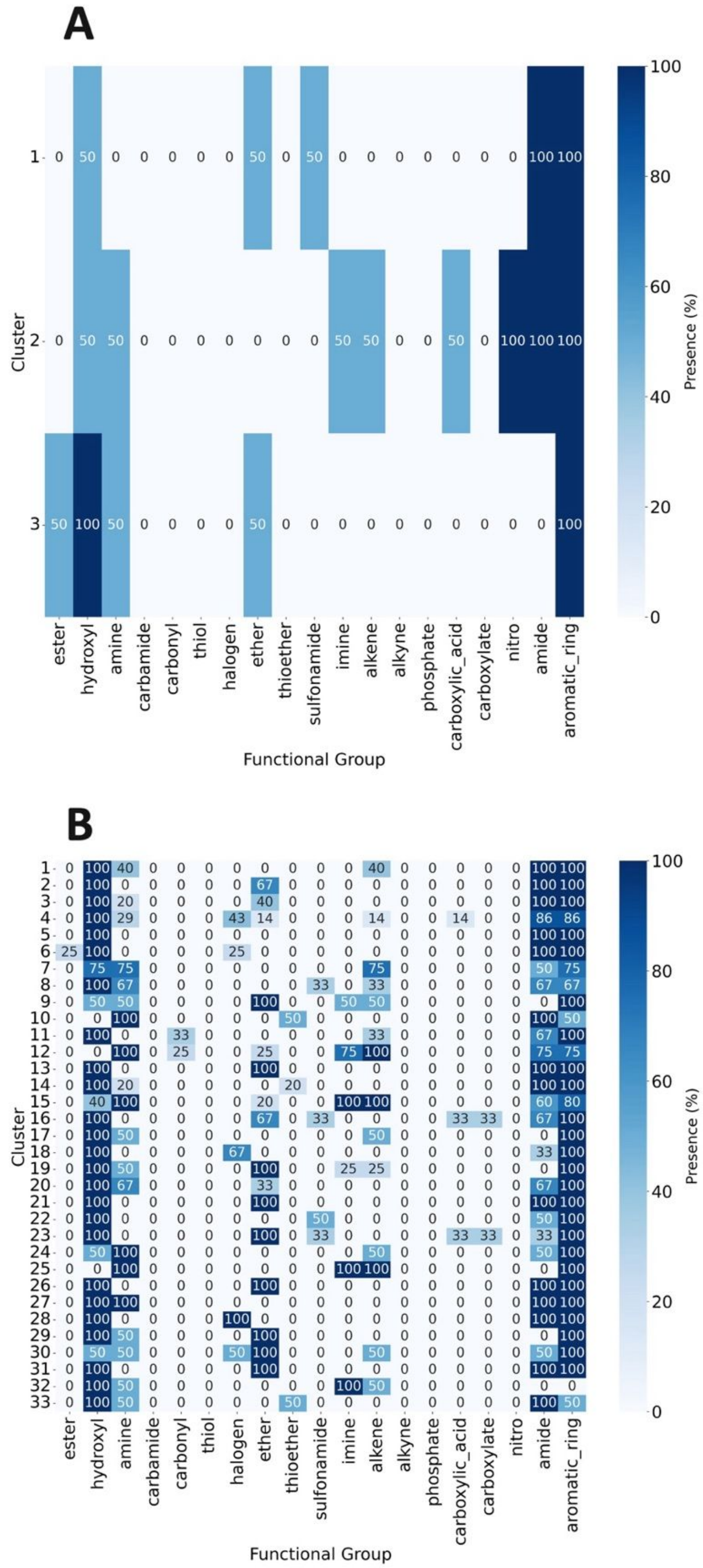

Figure S11. Heatmap showing the percentage occurrence of major functional groups within each cluster identified for α-amylase using the CHARMM FF. Clusters were derived from compounds obtained from (A) the Maybridge database and (B) the ChemBridge database. Values represent the proportion of ligands in each cluster containing the indicated functional group.

SUPPORTING TABLES

Table S1. List of FragMap names and the corresponding probe atoms.

FragMap Name	Generated from Probe Atom(s)
acec	Acetate carboxylate carbon
apolar	Generic apolar carbons (benc + prpc)
benc	Benzene carbons
dmeo	Dimethyl ether oxygen
forn	Formamide nitrogen
foro	Formamide oxygen
hbacc	Generic hydrogen bond acceptors (foro + dmeo + imin)
hbdon	Generic hydrogen bond donors (forn + iminh)
imin	Imidizole nitrogen (hydrogen bond acceptor)
iminh	Imidizole nitrogen (hydrogen bond donor)
mamn	Methylamine nitrogen
meoo	Methanol oxygen
prpc	Propane carbon
tipo	Water oxygen

Table S2. Overlap coefficients (OC) for FragMaps generated using conventional SILCS workflows with CHARMM and Drude FFs.

Fragment atom type	CHARMM			Drude		
	NtMGAM	CtMGAM	α-Amylase	NtMGAM	CtMGAM	α-Amylase
meoo	0.76	0.76	0.77	0.81	0.81	0.81
imin	0.77	0.76	0.77	0.74	0.73	0.74
iminh	0.77	0.76	0.77	0.74	0.72	0.73
foro	0.77	0.76	0.77	0.81	0.80	0.81
forn	0.77	0.76	0.77	0.81	0.80	0.81
forc	0.76	0.76	0.77	0.81	0.80	0.81
mamn	0.74	0.74	0.75	0.80	0.80	0.80
dmeo	0.76	0.76	0.77	0.80	0.80	0.81
benc	0.86	0.85	0.86	0.81	0.80	0.82
prpc	0.81	0.81	0.82	0.83	0.83	0.84
acec	0.75	0.74	0.75	0.74	0.71	0.71
gehc	0.85	0.85	0.85	0.80	0.79	0.80
tipo/swm4o	0.96	0.97	0.97	0.97	0.97	0.97

Table S3. Overlap coefficients (OC) for FragMaps generated using neutral-charged SILCS workflows with CHARMM and Drude FFs. OC values are reported separately for neutral and charged probe environments.

Fragment atom type	CHARMM			Drude		
	NtMGAM	CtMGAM	α-Amylase	NtMGAM	CtMGAM	α-Amylase
**Neutral**						
meoo	0.75	0.74	0.75	0.81	0.80	0.86
imin	0.75	0.74	0.76	0.75	0.74	0.80
iminh	0.75	0.74	0.76	0.75	0.74	0.80
foro	0.75	0.74	0.76	0.81	0.80	0.86
forn	0.75	0.74	0.77	0.81	0.80	0.86
forc	0.75	0.74	0.76	0.81	0.80	0.86
dmeo	0.75	0.75	0.77	0.81	0.81	0.87
benc	0.84	0.84	0.86	0.83	0.82	0.91
prpc	0.82	0.82	0.83	0.86	0.84	0.94
gehc	0.82	0.82	0.84	0.81	0.80	0.89
tipo/swm4o	0.98	0.97	0.97	0.98	0.98	0.99
**Charged**						
mamn	0.71	0.73	0.76	0.79	0.79	0.80
acec	0.77	0.77	0.78	0.78	0.76	0.76
aceo	0.83	0.83	0.83	0.83	0.81	0.80
tipo/swm4o	0.97	0.97	0.97	0.98	0.98	0.98

Table S4. Energetic and spatial properties of all candidate pharmacophore features generated for NtMGAM using the CHARMM additive and Drude polarizable force fields. The table reports feature type, FGFE, energetic rank, center coordinates, and tolerance radius.

CHARMM-derived features

Rank	Feature type	FGFE (kcal/mol)	Center X (Å)	Center Y (Å)	Center Z (Å)	Tolerance radius (Å)
1	Don	−96.17	−1.266	17.990	6.014	1.50
2	Don|Acc&!Ani	−94.96	−1.932	14.573	9.027	1.50
3	Cat&Don	−54.27	−9.030	19.103	2.647	1.50
4	Cat&Don	−52.91	−5.878	14.524	12.157	1.50
5	Cat&Don	−28.89	−3.224	21.986	2.580	1.50
6	Cat&Don	−22.13	−8.378	15.955	10.134	1.50
7	Cat&Don	−14.09	−11.092	17.041	9.734	1.50
8	Cat&Don	−11.38	0.422	13.155	5.134	1.50
9	Cat&Don	−8.54	−13.128	14.505	6.484	1.50
10	Aro	−5.11	−3.128	13.755	8.734	1.89
11	Aro	−4.99	1.872	19.755	5.984	1.97
12	Cat&Don	−2.27	1.622	8.755	11.734	0.87
13	Acc&!Ani	−1.15	−5.378	18.755	7.734	0.87

Drude-derived features

Rank	Feature type	FGFE (kcal/mol)	Center X (Å)	Center Y (Å)	Center Z (Å)	Tolerance radius (Å)
1	Don|Acc	−346.76	−5.193	16.278	10.108	1.50
2	Don	−71.62	−2.796	24.315	1.833	1.50
3	Don|Acc&!Ani	−71.21	−2.349	21.045	4.351	1.50
4	Don&!Cat|Acc	−66.90	−2.293	25.185	11.246	1.50
5	Aro|Hyd	−26.32	−2.944	13.278	16.444	2.50
6	Cat&Don	−18.53	−8.056	20.278	2.056	1.50
7	Cat&Don	−11.76	6.700	21.300	7.500	1.50
8	Aro	−11.43	−7.056	16.389	9.500	2.50
9	Don&!Cat	−10.90	−3.125	12.875	16.000	1.50
10	Cat&Don	−9.15	−5.500	25.000	1.750	1.50
11	Aro	−5.09	−10.250	14.750	13.250	2.28
12	Don	−4.88	3.840	19.170	7.500	1.50
13	Acc&!Ani	−3.83	2.833	21.500	6.167	1.50
14	Ani&Acc	−3.82	−7.000	25.500	11.500	1.22
15	Ani&Acc	−3.66	−9.500	24.000	10.500	1.22
16	Acc&!Ani	−3.60	−3.167	14.167	17.500	1.50
17	Cat&Don	−2.28	−10.500	20.500	2.500	0.87
18	Don&!Cat	−2.13	3.500	26.500	10.000	1.22
19	Cat&Don	−2.02	−3.500	7.500	5.500	0.87
20	Acc&!Ani	−1.26	−10.500	21.500	8.500	0.87

21	Don&!Cat	−1.23	−7.500	12.500	8.500	0.87
22	Acc&!Ani	−1.22	2.500	14.500	6.500	0.87

Table S5. Top ten ranked compounds from the Maybridge and ChemBridge databases for NtMGAM identified using CHARMM- and Drude-based pharmacophore screening. Compounds are ranked according to their LE scores calculated from SILCS-MC docking. LGFE and LE values (in kcal/mol and kcal/mol·atom, respectively) for known inhibitors (acarbose and miglitol) calculated using SILCS-MC pose refinement are included to enable direct comparison with the newly identified compounds.

**CHARMM**						**Drude**					
**Maybridge**			**ChemBridge**			**Maybridge**			**ChemBridge**		
**Ligand**	**LGFE**	**LE**	**Ligand**	**LGFE**	**LE**	**Ligand**	**LGFE**	**LE**	**Ligand**	**LGFE**	**LE**
SEW03510-3	-10.23	-0.93	61302850-3	-14.62	-0.73	NRB00489-1	-15.62	-0.71	57756402-1	-15.00	-0.79
RJC00059-1	-12.22	-0.87	82122986-2	-13.07	-0.69	HTS03187-1	-16.34	-0.63	48723263-2	-12.15	-0.72
RJC00691-3	-10.46	-0.87	88590213-1	-13.49	-0.64	SEW04046-1	-19.05	-0.62	32878085-2	-14.14	-0.71
JFD01241-1	-13.08	-0.82	70396043-2	-12.06	-0.64	S03783-1	-14.65	-0.61	13049217-3	-14.10	-0.70
SB01199-3	-8.99	-0.82	44512456-1	-12.05	-0.63	HTS13526-1	-18.41	-0.59	5182366-3	-11.93	-0.70
RJC00041-1	-13.06	-0.82	70396043-3	-12.05	-0.63	HTS13003-1	-16.31	-0.56	56422328-5	-16.02	-0.70
RJC03312-2	-10.58	-0.81	70396043-5	-11.76	-0.62	HTS12310-4	-16.72	-0.56	38663383-9	-13.89	-0.69
CD08263-2	-8.85	-0.80	85019669-2	-12.46	-0.59	SPB07794-1	-14.44	-0.56	16308800-1	-13.17	-0.69
CD05169-2	-8.80	-0.80	73194068-1	-11.60	-0.58	SPB07794-3	-14.37	-0.55	83165852-3	-13.84	-0.69
NRB04542-1	-12.63	-0.79	14065078-1	-9.76	-0.57	AW00809-2	-15.40	-0.55	88256668-4	-10.96	-0.68

**CHARMM**			**Drude**		
**Ligand**	**LGFE**	**LE**	**Ligand**	**LGFE**	**LE**
Miglitol	-6.74	-0.48	Miglitol	-6.81	-0.49
Acarbose	-4.14	-0.09	Acarbose	-9.98	-0.23

Table S6. Top ten ranked compounds from the Maybridge and ChemBridge databases for CtMGAM identified using CHARMM- and Drude-derived pharmacophore models. Compounds are ranked according to LE values obtained from SILCS-MC docking. LGFE and LE values (in kcal/mol and kcal/mol·atom, respectively) for known inhibitors (acarbose and miglitol) calculated using SILCS-MC pose refinement are included to enable direct comparison with the newly identified compounds.

CHARMM						Drude					
Maybridge			ChemBridge			Maybridge			ChemBridge		
Ligand	LGFE	LE	Ligand	LGFE	LE	Ligand	LGFE	LE	Ligand	LGFE	LE
KM08944-1	-10.05	-0.56	39178335-1	-11.31	-0.54	HTS12310-1	-17.46	-0.58	57665815-3	-14.43	-0.72
KM08943-1	-9.73	-0.51	16836628-7	-12.90	-0.54				64231628-8	-13.45	-0.67
KM08944-2	-8.77	-0.49	52576557-1	-9.19	-0.51				61462665-3	-13.77	-0.63
HTS09823-2	-13.02	-0.48	75631348-1	-10.59	-0.50				83259566-3	-15.00	-0.63
HTS12310-2	-13.64	-0.46	55607683-1	-10.06	-0.50				79769067-2	-13.00	-0.62
HTS09823-3	-11.30	-0.42	14599884-1	-9.80	-0.49				48929338-6	-14.94	-0.58
SPB07138-3	-8.71	-0.42	50789881-5	-9.80	-0.47				92355088-1	-11.85	-0.49
HTS11477-2	-10.10	-0.40	33278932-2	-10.71	-0.47				23896515-2	-12.62	-0.48
JFD01297-3	-8.82	-0.40	34547588-2	-9.42	-0.45				63104814-2	-13.35	-0.48
CD08052-2	-9.62	-0.40	54913770-2	-9.65	-0.44				91182239-4	-12.27	-0.47

CHARMM			Drude		
Ligand	LGFE	LE	Ligand	LGFE	LE
Acarbose	-9.33	-0.21	Acarbose	-9.93	-0.23
Miglitol	-6.83	-0.49	Miglitol	-6.42	-0.46

Table S7. Top ten ranked compounds from the Maybridge and ChemBridge databases for α-amylase identified using CHARMM- and Drude-derived pharmacophore models. Compounds are ranked according to ligand efficiency (LE) values obtained from SILCS-MC docking. LGFE and LE values (in kcal/mol and kcal/mol·atom, respectively) for a known inhibitor (myricetin) calculated using SILCS-MC pose refinement are included to enable direct comparison with the newly identified compounds.

**CHARMM**						**Drude**					
**Maybridge**			**ChemBridge**			**Maybridge**			**ChemBridge**		
**Ligand**	**LGFE**	**LE**	**Ligand**	**LGFE**	**LE**	**Ligand**	**LGFE**	**LE**	**Ligand**	**LGFE**	**LE**
S03367-1	-7.71	-0.48	81935788-2	-8.94	-0.47	NRB04426-1	-9.98	-0.24	34773317-1	-13.17	-0.53
KM08324-2	-9.06	-0.41	81935788-1	-8.75	-0.46				23507555-5	-12.53	-0.52
HTS00783-1	-8.71	-0.38	78054015-3	-12.42	-0.46				49450966-2	-13.33	-0.51
RJC03497-1	-9.58	-0.37	15700289-1	-9.51	-0.43						
AW00880-1	-10.31	-0.34	72141173-4	-9.82	-0.43						
HTS07123-1	-8.38	-0.34	51181516-2	-11.07	-0.43						
HTS07122-1	-8.42	-0.31	50789546-2	-8.53	-0.43						
HTS11989-2	-8.97	-0.26	44846457-1	-10.13	-0.42						
			48338115-2	-11.09	-0.41						
			93026738-4	-11.08	-0.41						
**CHARMM**						**Drude**					
**Ligand**	**LGFE**			**LE**		**Ligand**	**LGFE**			**LE**	
Myricetin	-4.05			-0.18		Acarbose	-2.85			-0.12	

Table S8. Representative scaffold structures and corresponding statistical parameters obtained from Bemis–Murcko clustering for the clusters with the most favorable ligand efficiency scores in the N-terminal domain of maltase-glucoamylase, based on Maybridge and ChemBridge virtual screening hits.

Model and Database	Scaffold Structure	Number of Compounds	Mean LE	Median LE	Q1	Q3	IQR
CHARMM/ Maybridge	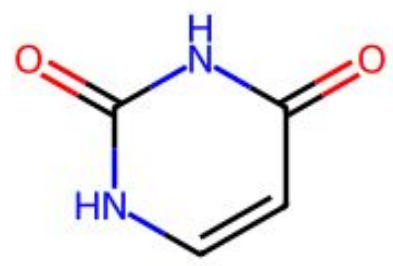	5	-0.74	-0.77	-0.78	-0.74	0.04
CHARMM/ ChemBridge	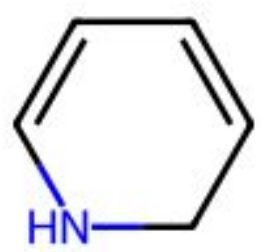	2	-0.56	-0.56	-0.56	-0.55	0.01
Drude/ ChemBridge	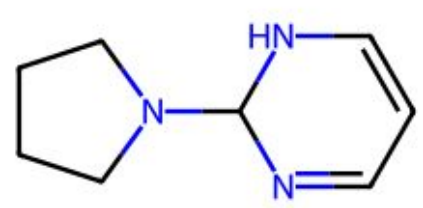	3	-0.68	-0.67	-0.69	-0.67	0.02

Table S9. Representative scaffold structures and corresponding statistical parameters obtained from Bemis–Murcko clustering for the clusters with the most favorable ligand efficiency scores in CtMGAM, based on Maybridge and ChemBridge virtual screening hits.

Model and Database	Scaffold Structure	Number of Compounds	Mean LE	Median LE	Q1	Q3	IQR
CHARMM/ Maybridge	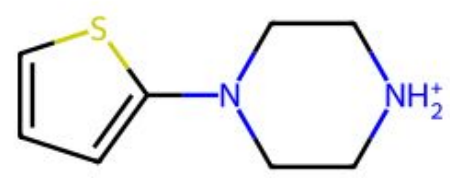	2	-0.54	-0.54	-0.55	-0.52	
CHARMM/ ChemBridge	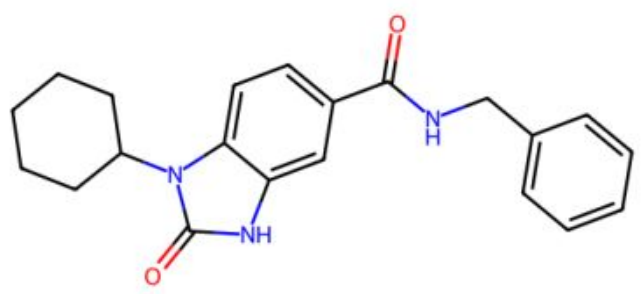	2	-0.32	-0.32	-0.32	-0.32	0.003

Table S10. Representative scaffold structures and corresponding statistical parameters obtained from Bemis–Murcko clustering for the clusters with the most favorable ligand efficiency scores in the α-Amylase, based on Maybridge and ChemBridge virtual screening hits.

Model and Database	Scaffold Structure	Number of Compounds	Mean LE	Median LE	Q1	Q3	IQR
CHARMM/ Maybridge	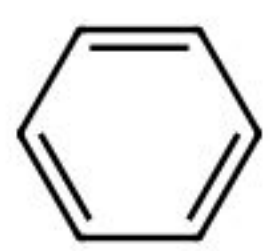	2	-0.45	-0.45	-0.46	-0.43	0.04
CHARMM/ ChemBridge	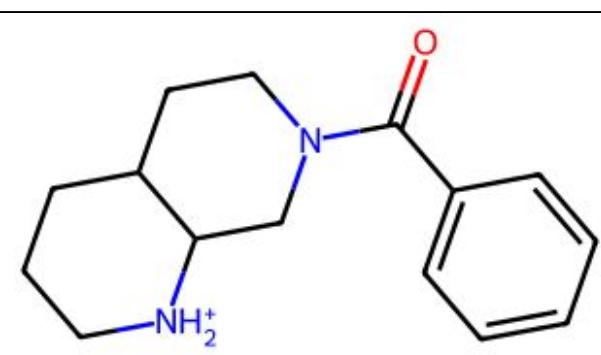	7	-0.33	-0.33	-0.35	-0.32	0.02

References

(1) Goel, H.; Hazel, A.; Yu, W.; Jo, S.; MacKerell, A. D. Application of site-identification by ligand competitive saturation in computer-aided drug design. *New J. Chem.* **2022**, *46* (3), 919-932.

(2) Guvench, O.; MacKerell Jr, A. D. Computational fragment-based binding site identification by ligand competitive saturation. *PLoS Comput. Biol.* **2009**, *5* (7), e1000435.

(3) Michel, H. M.; Brown, A. M.; MacKerell Jr, A. D.; Lemkul, J. A. Drude SILCS-Nucleic: Harnessing Explicit Electronic Polarization in Targeting RNA and DNA for Drug Design. *bioRxiv* **2025**, 2025.2012. 2020.695737.

(4) Ustach, V. D.; Lakkaraju, S. K.; Jo, S.; Yu, W.; Jiang, W.; MacKerell Jr, A. D. Optimization and evaluation of site-identification by ligand competitive saturation (SILCS) as a tool for target-based ligand optimization. *J. Chem. Inf. Model.* **2019**, *59* (6), 3018-3035.

(5) Vanommeslaeghe, K.; Hatcher, E.; Acharya, C.; Kundu, S.; Zhong, S.; Shim, J.; Darian, E.; Guvench, O.; Lopes, P.; Vorobyov, I. CHARMM general force field: A force field for drug-like molecules compatible with the CHARMM all-atom additive biological force fields. *J. Comput. Chem.* **2010**, *31* (4), 671-690.

(6) Faller, C. E.; Raman, E. P.; MacKerell Jr, A. D.; Guvench, O. Site Identification by Ligand Competitive Saturation (SILCS) simulations for fragment-based drug design. In *Fragment-based methods in drug discovery*, Springer, 2015; pp 75-87.
